# A standardized validity assessment protocol for physiological signals from wearable technology: Methodological underpinnings and an application to the E4 biosensor

**DOI:** 10.3758/s13428-019-01263-9

**Published:** 2019-07-09

**Authors:** Hendrika G. van Lier, Marcel E. Pieterse, Ainara Garde, Marloes G. Postel, Hein A. de Haan, Miriam M. R. Vollenbroek-Hutten, Jan Maarten Schraagen, Matthijs L. Noordzij

**Affiliations:** 1 Department of Cognitive Psychology and Ergonomics, University of Twente, Enschede, The Netherlands; 2 Department of Psychology, Health and Technology, University of Twente, Enschede, The Netherlands; 3 Department of Biomedical Signals and Systems, Telemedicine Group, University of Twente, Enschede, The Netherlands; 4 Tactus Addiction Treatment, Enschede, The Netherlands; 5 Ziekenhuis Groep Twente, ZGT Academy, Almelo, The Netherlands; 6 Netherlands Organization for Applied Scientific Research (TNO), The Hague, Netherlands

**Keywords:** Electrodermal activity, Skin conductance, Photoplethysmogram, Heart rate, Stress, Habituation effect

## Abstract

Wearable physiological measurement devices for ambulatory research with novel sensing technology are introduced with ever increasing frequency, requiring fast, standardized, and rigorous validation of the physiological signals measured by these devices and their derived parameters. At present, there is a lack of consensus on a standardized protocol or framework with which to test the validity of this new technology, leading to the use of various (often unfit) methods. This study introduces a comprehensive validity assessment protocol for physiological signals (electrodermal activity and cardiovascular activity) and investigates the validity of the E4 wearable (an example of such a new device) on the three levels proposed by the protocol: (1) the signal level, with a cross-correlation; (2) the parameter level, with Bland–Altman plots; and (3) the event level, with the detection of physiological changes due to external stressor levels via event difference plots. The results of the protocol show that the E4 wearable is valid for heart rate, RMSSD, and *SD* at the parameter and event levels, and for the total amplitude of skin conductance responses at the event level when studying strong sustained stressors. These findings are in line with the prior literature and demonstrate the applicability of the protocol. The validity assessment protocol proposed in this study provides a comprehensive, standardized, and feasible method for assessment of the quality of physiological data coming from new wearable (sensor) technology aimed at ambulatory research.

Electrodermal activity (EDA) and cardiovascular activity (CVA) have been used as measures of various constructs linked to the autonomic nervous system (Sawada, Tanaka, & Yamakoshi, 2001), such as stress. Current development of wearable devices measuring EDA and CVA wirelessly widens the range of (research) applications (Poh, Swenson, & Picard, 2010; Torniainen, Cowley, Henelius, Lukander, & Pakarinen, 2015), especially to ambulatory assessment (Wilhelm, Perrez, & Pawlik, 2012). Ambulatory assessment is a computer-assisted method for monitoring participants while they carry out their daily activities (Trull & Ebner-Priemer, 2013). Due to the novel nature of the wearables, rigorous validation of the physiological signals measured by these devices and their derived parameters is required for the purpose of research. Wearable devices are relatively rapidly replaced by newer alternatives. The market is quite diverse and various research and commercial devices are available, of which the quality is uncertain. Therefore, there is an urgent need for a systematic and comprehensive, yet fast and easily replicable, validity assessment protocol However, such a standardized protocol is currently not available, so researchers carrying out a validity assessment study need to make their own judgment call or spend a lot of time investigating the method options (Kottner et al., 2011). Additionally, for the variety of methods available no clear criteria are defined to evaluate the validity of a new device. The aim of this article is therefore to propose a standardized validity assessment protocol with standardized techniques for analyses and decision criteria to assess EDA and CVA wearables. Additionally, the proposed protocol will be applied to a state of the art research wearable.

Currently, validation assessment of EDA an CVA wearables often come to inconclusive and incomparable inferences for three reasons: (1) the use of different (sometimes inappropriate) statistical methods (Zaki, Bulgiba, Ismail, & Ismail, 2012), (2) evaluation on different variable levels, and (3) the lack of decision criteria to determine validity (Giavarina, 2015). The use of different and sometimes even inappropriate statistical methods (Watson & Petrie, 2010) applied to different types of variables with inconclusive results makes comparison between validation studies difficult and makes choosing proper methodology to do new validation studies seemingly arbitrary. The effect of these three issues in prior research are discussed below.

First, researchers often analyze the validity with (different) statistical methods (Zaki, Bulgiba, Ismail, & Ismail, 2012), some of which are inappropriate for validation assessment. Sartor et al. (2018) remarked that for heart rate sensors validation studies are often inconclusive, due to methodological issues. An example of such an inappropriate statistical method is intraclass correlation when comparing parameters, like number of skin conductance responses.

Second, researchers compare different variables of interest leading to incomparable studies even if results are conclusive. Sometimes they assess whether a wearable can detect an underlying construct like stress, at other times researchers evaluate whether the device produces a comparable result to a reference device (RD) for a parameter (e.g., heart rate [HR]), regardless of the context.

Third, validity assessment of wearable devices is often performed for either researchers’ own prototypes (e.g., Poh et al., 2010; Torniainen et al., 2015), commercial devices (Sartor, Papini, Cox, & Cleland, 2018), or for unconventional electrode placement (e.g., on the wrist instead of the fingers) often used for wearables (e.g., Payne, Schell, & Dawson, 2016; van Dooren, de Vries, & Janssen, 2012). The latter two often arrived at indefinite conclusions about the use of wearables, since no criteria are defined a priori to decide whether the device is valid. For example, Payne et al. concluded that the wrist was found not to be a viable replacement of the finger location, which is generally used. This is because the wrist produces fewer responses than the fingers and therefore has a low coherence with the fingers. However, Payne et al. still argued that they do see a role for the wrist placement in the ambulatory setting (under certain conditions). Whether the wrist is sensible to use in these settings remains inconclusive. It is therefore important that not only a standardized method of assessment is chosen upfront, but also decision criteria on when a device is valid or not.

Other researcher have begun to advocate for more structured and systematic validation protocols. For example, Kayhan et al. (2018) set out to provide a comprehensive protocol for the validation of wearable sociometric badges (i.e., a tool to study interpersonal processes). In this article, we offer a standardized protocol including decision criteria to assess the validity of a wearable device by comparing it to a reference device (RD). We aim to not just propose a set of guidelines for validation, but a standardized protocol using proper statistical methods and decision criteria. We aim for a protocol that allows for standardization for a variety of wearables and contexts of intended use.

From prior research we identified three types of variable levels used: the *signal*, *parameter* and *event level*. We propose a standardization of which statistical methods to use in order to analyze these levels with corresponding decision criteria. The first level proposed, the *signal level*, is the most direct form of comparison. It assesses the extent to which new devices are capable of generating roughly the same raw data as the established hardware. The *parameter level* is relevant to determine whether a new device produces physiological parameters (e.g., HR) for each individual similar to the RD. The *event level* is the assessment of the target of the study; a comparison is made on ability to significantly detect an event(s) via a group mean, for example a response to a stressor, with both devices and compare between them. In the next sections, the levels, standardized methods proposed, and decision criteria chosen will be explained in detail. In addition, a first complete overview of the protocol with the decision criteria for each level is already provided in Fig. 1.

**Fig. 1:**
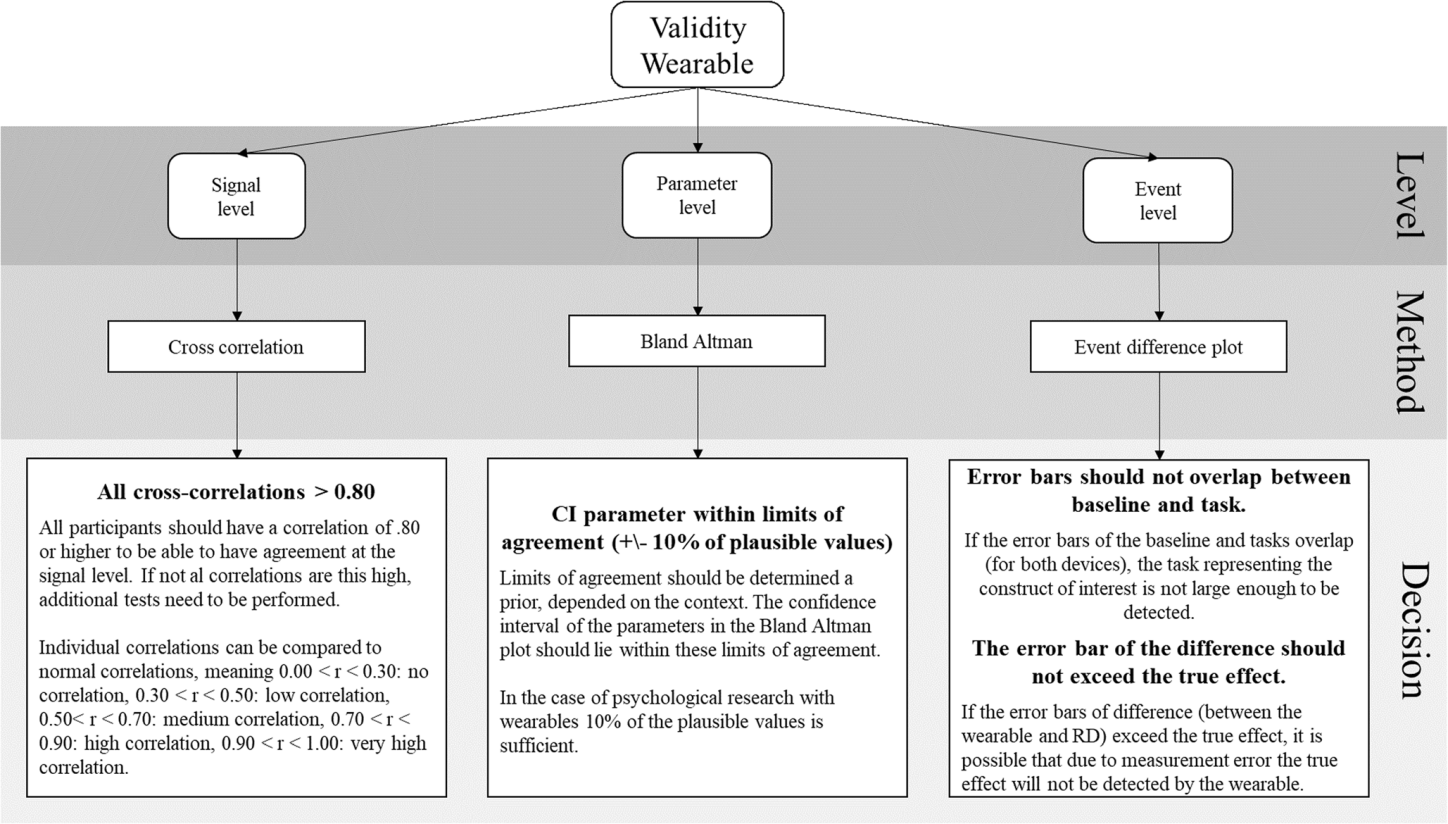
Overview of the complete protocol and the decisions that can be made from it concerning the validity of a device

## Signal level

The standard process of assessing the concurrent validity of a device is by comparing the measurement of the device to the RD, sometimes also called agreement (Kottner et al., 2011). Because the RD is accepted as validly measuring a certain construct, it is assumed that a device that produces similar signals is also valid. The question on the signal level is whether the error introduced by using the wearable device lies within acceptable boundaries from the signal retrieved by the RD.

For EDA, the RD is provided by measuring skin conductance (SC), measured in microsiemens, at the intermediate phalanges of the ring and index finger (Boucsein, 2012), whereas the wearable often measures SC with electrodes located at the wrist. There are some differences between these two locations. The wrist has fewer sweat glands than the fingers (Boucsein, 2012), and some argue that at the wrist emotional sweating is more evident (Wilke, Martin, Terstegen, & Biel, 2007). Therefore, finding full agreement on signal level (raw data) between the devices is highly unlikely. However, this level is relevant to be included in the protocol for two purposes. First, to accommodate researchers who are interested in using the raw data of the wearable. Second, when a golden standard for wearable devices becomes available, agreement on this signal level is likely, and when there is agreement on this level, assessment on other levels is unnecessary.

**Fig. 2:**
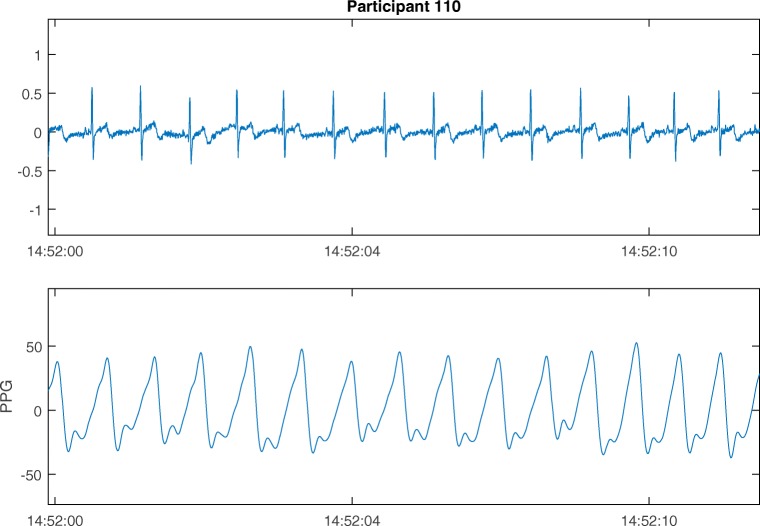
Electrocardiograph (ECG) and photoplethysmograph (PPG) signals of a participant within the present study

For CVA the RD signal is often retrieved with electrodes at the chest with electrocardiography (ECG), whereas wearable devices typically are located at the wrist and use photoplethysmography (PPG). PPG is a light-based technique used to acquire CVA. The amount of light reflected in the blood at each time can be used to determine peaks in the blood flow (Shelley & Shelley, 2001). The number of peaks per minute are the HR. HR is expected to be similar at the chest and the wrist. However, due to the different techniques used the signal produced by the measures is different. Examples of both the PPG and ECG signals are presented in Fig. 2.

The ECG shows a well-known QRS wave with a small dent prior to a steep peak, followed again by a dent. The PPG signal has less steepness in its peaks. A comparison at the signal level will not be useful, since it would be unclear which part of the introduced error was from the use of a different technique and which was introduced by the wearable. Therefore, direct comparison between these two signals at the signal level is not possible.

### Standardized statistical method for the validity assessment at signal level

We recommend to use cross-correlation to compare the wearable to a RD at the signal level. Cross-correlation is an often used measure to determine the similarity of two time series as a function of the displacement of one relative to the other (McCleary, Hay, Meidinger, & McDowall, 1980). Cross-correlation is a generalization of Pearson’s correlation (as used by, e.g., Poh et al., 2010, who found a correlation between .93 and .99 for a wearable and RD for EDA) to the displacement in time, through which systematic time delays between the two signals can be detected. Watson and Petrie (2010) mention that a correlation could miss a mean bias between two measures. It is therefore advised by Giavarina (2015) to test for the mean difference between the two signals if the cross-correlation is high. If a mean difference is found, this bias can easily be adjusted by centering the data around this mean. Additionally, a systematic difference in the variance can also be corrected. Other methods used at the signal level are visual inspections to compare the raw signals, see, for example, Ollander, Godin, Campagne, and Charbonnier (2016) and Poh et al. (2010). Ollander et al. found no visual similarity between two EDA signals. However, no clear statistical inferences can be drawn from visual inspection and we therefore do not recommend to rely on this particular method in isolation.

### Decision criteria for validity at signal level

Equal to correlation, cross-correlation lies between – 1 and 1. Similarly, the cross-correlations can be interpreted the same as standard correlations, meaning that .00 < *r* < .19 is a very weak correlation, .20 < *r* < .39 a weak correlation, .40 < *r* < .59 a moderate correlation, .60 < *r* < .79 a strong correlation, and .80 < *r* < 1.00 a very high correlation (Evans, 1996). The wearable can be determined to be valid if the cross-correlations are higher than .80 for each of the participants, meaning a very high correlation according to (Evans, 1996). Only a very strong correlation can support validity on the signal level, because for lower correlations the source of incomplete overlap between the two signals is hard to detect and therefore hard to adjust for. This high standard of agreement is probably never met due to the use of different measurement techniques or placement of the wearable, as discussed before. Additional tests should therefore be performed at both the parameter and event level in order to investigate the consequences of the lower cross-correlations at this level.

## Parameter level

Parameters are aggregated values over a timeframe for a person. The wearable is expected to be valid when these parameters are similar within certain boundaries, and as with the signal level, there will always be some measurement error. Multiple parameters retrieved from both the EDA and CVA signals could be used to compare the wearable with a RD, as is discussed in Box 1.

**Box 1** Commonly used parameters retrieved from EDA and CVA signals.

**Table:** 

For EDA, common parameters (Boucsein, 2012) are: - skin conductance level (SCL) - number of skin conductance responses (SCRs) - amplitude of the skin conductance responses (S-AMPL) And for CVA two types of parameters (or features) are extracted, namely from the frequency domain and from the time domain (for more information, see Berntson, Quigley, & Lozano, 2007). From the frequency domain: - (normalized) low frequency - (normalized) high frequency - ratio between low and high frequencies And for the time domain: - mean RR interval (RR interval is the time between two measured heart beats) or heart rate (HR) - standard deviation (*SD*) of the RR interval - root mean square of successive differences (RMSSD) of the RR interval. There are multiple variations on the RMSSD available, such as the standard deviation of the beat-to-beat or NN interval (SDNN) or the standard deviation of successive differences (SDSD).	

### Standardized statistical method for the validity assessment at parameter level

We recommend comparing the parameters retrieved from the wearable to the RD with Bland–Altman plots. Using the Bland–Altman plot is at this level better than using for example a Pearson’s correlation (e.g., Payne et al., 2016; Van Dooren et al., 2012), because Pearson’s correlation is not sensitive to a linear movement of all the observations on one of the scales (Bland & Altman, 1986). Both Payne et al. and Van Dooren et al. found that the wrist presented lower responses on multiple EDA parameters than the fingers, which would lead to a mean bias. Payne et al. additionally determined a concordance measure to compare the agreement between multiple sites. They found that in only 30% of the cases when an SCR occurred at the fingers was there a simultaneous occurrence of an SCR at the wrist. However, during the stress task this percentage was as high as 72%. Payne and colleagues also found lower responses for the wrist on multiple parameters. These results indicate that there might be substantial differences in (registering) physiological measures between the wrist and fingers. We do not use the concordance measure, since this is a very strict measure and only determines underestimation and cannot determine overestimation of SCRs by the wearable. The Bland–Altman plot can determine both over and underestimation of the wearable and boundaries can be chosen that are as strict as needed for the intended context of use.

An additional benefit of the Bland–Altman plot is that it not only looks at the mean overall difference among participants, but also takes into account the difference for each participant. Therefore it can determine multiple other systematic biases apart from fixed mean differences, which is the only bias an analysis of variance (ANOVA; as used by Nunan et al., 2008, and Payne et al., 2016) can detect. Even though the intraclass correlation (ICC) also tries to overcome problems related to correlation and ANOVA, Zaki, Bulgiba, Ismail, and Ismail (2012) are quite critical toward the ICC for multiple reasons: The ICC ignores ordering and treats both methods as a random sample from a population of methods, not two specific methods. Another issue is that the ICC depends on the range of the data, meaning that if the variance between participants is high, the value of ICC will certainly appear to be high. Although the use of the ICC seems to be popular, the appropriateness of this method to assess agreement is questionable. The Bland–Altman does not have these problems and as such is the most appropriate method to use (Zaki et al., 2012). A last benefit of the Bland–Altman plot is that missing data and nonnormality are visible and not hidden as they can be in data with outcome statistics. Therefore making inferences without checking the assumptions is not possible.

### Decision criteria for validity at the parameter level

Bland–Altman plots are often only implemented as a visual representation of the data instead of testing the agreement. From these Bland–Altman plots multiple systematic biases can be detected, among others mean shift and difference in variance (Giavarina, 2015). If a systematic bias is found in the Bland–Altman plot, this can either be further explored or corrected for. A mean shift bias shows, for example, that the wearable over- or underestimated all values. However, when there is no systematic bias, it is still not certain that the wearable agrees with the RD. Only when a priori acceptable boundaries are defined related to the maximum difference between the parameters retrieved from the RD and the wearable, can a judgment on the level of agreement be made (Giavarina, 2015). For the majority of measures such boundaries are a matter of discussion and context. This context can be formed by clinical necessity (e.g., precision of measurements needed to establish a certain medical condition), biological considerations (e.g., heritage or gender; O’Neal, Chen, Nazarian, & Soliman, 2016), the type of research, the time over which to aggregate (minutes vs. hours), the parameter of interest (e.g., HR), and possibly other criteria. A good validity assessment protocol makes this discussion and context explicit and provides a reasoned and transparent choice, which other researchers can replicate or adapt.

For some parameters, there are standards for this limit of acceptable error. The recommendations for heart rate equipment are a 5-beat-per-minute (BPM) difference or 10%, whichever is greater, as compared to RDs as defined by the Association for Advancement of Medical Instrumentation (2003). Since the biologically plausible values for instantaneous HR during a seated task are between 60 and 110 bpm, the boundaries of the Bland–Altman are ± 5 bpm, which is this 10%. We therefore recommend extending this 10% difference to the other CVA and EDA variables as well, even though EDA variables are different from CVA in nature. CVA data are for example highly periodic and EDA parameters like number of SCR’s are more event like. However, the AAMI proposed a limit that is rather strict since it is used for medical cardiac devices, therefore extending this limit to EDA is rather too strict than too tolerant, which is a good starting point for this newly devised protocol. The plausible values for the number of SCRs lie between 0 and 20 per minute (Dawson, Schell, & Filion, 2007) when using a threshold of 0.01. Therefore, the SCRs retrieved for a person with the wearable should lie within two SCRs from the number retrieved with the RD. If the parameter retrieved with the wearable lies within the limits of agreement, this suggests that the parameter is validly measured by the wearable.

Note that the Bland–Altman plot aggregates data over a person, meaning that an overestimation at the baseline could be compensated by an underestimation during a stress task. We therefore always recommend assessing the event level, even when the parameter level indicates agreement on all parameters.

## Event level

The raw EDA signal level is not often used in (ambulatory) research and the raw CVA signal probably never. Sometimes an aggregated value (parameter) for a person retrieved from the signal (like heart rate) is of interest, but most often, physiological reactions related to phenomena like stress or aggression are the real target. Sartor et al. (2018) suggest to test the device in the context of the intended use. Whether the parameter can validly be measured is then dependent on whether the physiological changes associated to the phenomenon of interest can validly be detected.

Within the present study *responses to short-term stressors* is chosen as construct of interest. To assess the wearable on a spectrum of short stressors, we recommend to apply one longer (30 s), strong, social stressor, the (slightly adapted) sing-a-song stress test (Brouwer et al., 2018), and one task with multiple smaller environmental stressors (a noise task; Bali & Jaggi, 2015). The first task resembles an experience an individual could have in daily life, the latter is a laboratory oriented task that should produce a distinct pattern in the EDA signal, namely the habituation effect in the amplitudes of the SCRs. The first is found to increase both HR and SC. The second task is not expected to increase HR as measured by the RD and can therefore be used to test the event level step of the protocol. If this effect is indeed not recorded by the RD in the CVA signal, the protocol should identify this as inconclusive. We propose these two short stressors, since these have a clear confirmed expected effect and are relatively easy and quick to use in an experimental setting. Note that by choosing short stressors the frequency domain of heart rate data cannot be evaluated, since these need longer periods to be determined reliably.

### Standardized statistical method for the validity assessment at event level

We recommend using a visualization of the wearable and the RD, first separately and then combined as difference scores preceding and during the stressor events. Since correlations (e.g., Payne et al., 2016; Poh et al., 2010) are argued to be improper methods of comparison due to possible undetected scale shifts (Bland & Altman, 1986), we use a visualization of the signals caused by the stressor and an assessment of the agreement of the two signals retrieved by the devices during this stressor. A review by Jennings and Gianaros (2007) showed that almost 85% of the articles in psychophysiology used a (repeated measures) ANOVA. We recommend use two types of visualizations instead, since the same inferences can be drawn but they will be easier to interpret for a broader audience than the repeated measures ANOVA. Obviously, we could use more sophisticated methods to analyze stress responses. However, we aim to keep the analysis executable by researchers and clinicians. Additionally, at the event level the focus is on robust effects that should show up in a less sophisticated analysis.

The first visualization is a separate line plot for the RD and the wearable with the mean and the *SE* per task (baseline, stressor etc.) and a line through the zero *y*-axis, where each individual is represented in a line (see Fig. 9 in the Results for clarification). From this plot the existence of the effect of the stressor (or other event) can be determined. The second proposed visualization is a line plot in which the differences between the wearable and the RD for each person are represented with a line, with the mean and the *SE* per task and a line through the zero *y*-axis (see Fig. 10 in the Results for clarification). From this plot, the lack of effect from the use of the wearable relative to the RD can be observed. However, since the lack of effect is not the same as agreement between the two devices a Bland–Altman like approach will be used. Instead of plotting the mean per person at the y-axis, the group mean per task will be used. Boundaries are proposed between which the mean and corresponding standard error should lie.

To determine the difference between the wearable and RD, a detectable stressor needs to be present, such that it should elevate a response that is significantly higher than a predefined baseline. Payne et al. (2016) did not find a significant stress response for either the wrist or the finger placement, leading to indefinite conclusions on placement. Payne and colleagues argued that the wrist has potential only in an ambulatory setting where a stronger stressor (than the math and the International Affective Picture System task they used) is present. Van Dooren et al. (2012) presented a high stressor and found heightened skin conductance responsiveness (SCL, SCRs, and S-AMPL) with a repeated measures ANOVA to emotional film clips by both the fingers and the wrist. If the stressor is not detected, no further inferences about the validity of the wearable to detect this stressor similarly to the RD can be made.

### Decision criteria for validity at the event level

For the first visualization, if the error bars of the baseline and stressor tasks overlap (for the RD), the signal representing the construct of interest is not large enough to be detected. Only when the RD can detect the construct of interest, can the validity of the wearable be further assessed. The same assessment needs to be made to verify whether the wearable also shows the difference between baseline and task. If the wearable does not detect the stressor even though the RD does detect it, the wearable is not valid to detect the stressor and no further analysis needs to be made for this level.

However, when both devices detect the effect, a second visualization needs to be made, in which the error bars of the difference between the wearable and RD should first of all overlap with the zero axis. If the error bar for a task does not cross the zero axis, there is a significant difference between the RD and the wearable. This means that the wearable is measuring something different than the RD or detects false positives or negatives. However, as mentioned before if they do overlap this is no sufficient evidence of agreement between the wearables. Therefore, a Bland–Altman-like analysis is proposed. An a priori acceptable range that the group mean may differ from the RD is chosen. If the difference mean and corresponding standard error lie between the a priori defined boundaries, the devices show agreement for the tasks.

It would be optimal to determine a boundary based on theoretical tolerance of the wearable, however this will not always be possible. Therefore, we recommend a boundary based on the effect detected by the RD. We recommend that the boundary is the “true” effect of the variable of interest, this reference effect is the difference between the baseline and the task with the variable of interest measured by the RD. This is a proper boundary, since this entails that the wearable always finds the effect from the variable of interest even if the mean found is biased due to measurement error. A smaller boundary is possible when this can be theoretically founded, however the reference effect (by the variable of interest) is large enough to allow for some difference between the devices. If the wearable exceeds this boundary, it will not be able to distinguish any difference between the true baseline and the task, meaning that a baseline measure is always needed while using the wearable.

## Applying the validity assessment protocol: E4 wearable

**Fig. 3:**
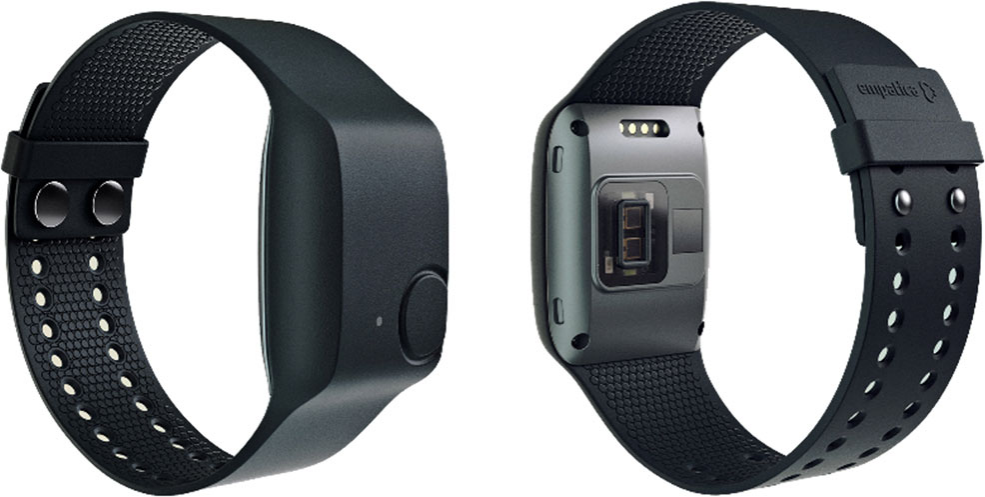
E4 wristband—Empatica, reproduced with permission

For research purposes, many wearable devices exist to measure CVA accurately (Giles, Draper, & Neil, 2016). However, for measuring EDA, only a small number of wireless devices are currently available (Majumder, Mondal, & Deen, 2017), and when needing to collect data from one wearable for both CVA and EDA simultaneously, the number of devices is even lower (Pantelopoulos & Bourbakis, 2010). In the present study, we tested the E4 wristband by Empatica (see Fig. 3), a wearable biosensor that measures both CVA and EDA signals. We will use the E4 wearable as an application example to illustrate our validity assessment protocol.

Preliminary studies on the validity of the PPG sensors of the E4 wearable indicated comparable data quality in 85% of the data (of seven participants) for the CVA between E4 wearable and a RD (McCarthy, Pradhan, Redpath, & Adler, 2016). The RD performed better in only 5% of the cases. Ollander, Godin, Campagne, and Charbonnier (2016) also performed a small sample study (*n* = 7) and found, at the signal level, significant loss in terms of detected heartbeats, especially when performing a task. However, retrieved parameters like the mean and the standard deviation of HR were well estimated. At the event level, they found comparable sensitivity between the E4 wearable and RD for participants performing the Trier Social Stress Task (on mean and *SD* of HR). Ollander et al. also visually compared the EDA signal level and found no visual resemblance between the EDA signal for the E4 wearable and the RD. They did not compare at the parameter level for EDA. At the event level, they found that the phasic driver showed a higher physiological reaction to stressors (Trier Social Stress Task) than the RD. However, whether this higher physiological reaction is significantly different is not possible to determine via the method they used (ROC curve). The same holds for the finding of comparable sensitivity for the CVA data. Zheng and Poon (2016) performed a 20-h experiment on one participant and found a strong correlation between activity and multiple cardiovascular parameters, showing a confound of physical activity with psychological activity. For the EDA measures, 78% of the data was classified as artifact and no further analysis was performed. Currently, no other validity studies of the E4 wearable have been performed. On the basis of the variability in methods, low sample size, and inconclusive findings, further validation of the E4 wearable is required. Therefore, this wearable biosensor poses a relevant case to test our proposed validity assessment protocol.

## Method

The aim of this article is to propose a validity assessment protocol and assess this protocol using the E4 wearable, which at the time this study was conducted was the only wearable wrist-worn device measuring both EDA and CVA. The experiment used to assess the validity of the E4 wearable is described below. The data collected in this study can be requested from the first author. The code used to analyze this data can be found at https://github.com/HendrikavanLier/validityassessmentprotocol.git.

### Participants

We determined that a sample of 55 persons is sufficient to establish 90% power on the three levels in this experimental procedure. Since this protocol uses multiple techniques, the power analysis was performed on all levels. A detailed overview of this power analysis can be found in the Appendix. We assumed that multiple participants would have unusable data, therefore more participants were recruited than the 55 suggested. Seventy-seven participants (42 male, 35 female; mean age: 23.0 years, *SD* = 4.0) took part in the experiment. Participants were informed that they would participate in a validity assessment study for the E4 wearable, not that the tasks were related to stress. Participants with any health issues such as heart diseases or epilepsy were excluded from the study. The participants were students at the University of Twente or students at the Academy of Pop Music and Media Music. Students of the University of Twente participated in exchange for course credits in this study. Additionally, one randomly selected participant was rewarded with a gift certificate that was used as an incentive to participate. Informed consent was obtained from all individual participants included in the study. The Ethics Committee of the University of Twente approved that the study is in accordance with the standards listed in the faculties’ Protocol about Ethics and Research.

### Design

**Fig. 4:**
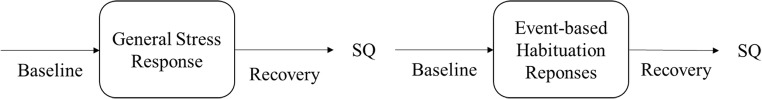
Experiment design. General Stress Response indicates the sing-a-song-stress test, and Event-Based Habituation Responses shows the noise task. SQ = stress questionnaire

Two stress tasks were presented to the participants: first a slightly adapted version (Brouwer et al., 2018) of the sing-a-song-stress test (Brouwer & Hogervorst, 2014), followed by a noise task. The design of the experiment is shown in Fig. 4.

### Sing a song stress task (general stress response)

The sing-a-song stress test (SSST) was used as a strong social stressor. The SSST is a fast social stress test that can be performed with one researcher in less than 10 min yielding a high stress response (Brouwer et al., 2018), making it a more accessible alternative to the Trier Social Stress Test (Kirschbaum, Pirke, & Hellhammer, 1993), which is an effective but labor-intensive task. Given that the aim of this study was to propose a quick and feasible validation, the SSST is preferred over the Trier Social Stress Test. The SSST was found to be inducing most stress when compared to several other stressors, both social, environmental and cognitive (Egilmez et al., 2017). For heart rate, Brouwer and Hogervorst found an increase of 15.3 bpm, which is approximately 21% (determined from the figure). In addition, for EDA, Brouwer and Hogervorst found an increase of 10.9 μS (approximately 55% increase).

During the SSST, the participants were seated in front of a screen that presented four consecutive neutral messages in time intervals of 40 s. Next, the participants were informed that they had to sing a song later on, and that they should now start to think about a song they could sing. After the anticipation period of 30 s, the final message requested the participant to start singing and not to stop for 30 s. Then, the participants were again asked to sit quietly and focus on their breathing for 2 min during the recovery period.

### Noise task (event-based habituation responses)

The second stressor used was an environmental stressor. Noise is a pervasive and influential source of stress (Szalma & Hancock, 2011) and a commonly used form of an environmental stressor (Bali & Jaggi, 2015). In this experiment, noise was applied via headphones. The participants had to listen to the noise in the form of 1000-Hz beep sounds, which lasted for 200 ms each and were approximately 75 dB loud. A physiological response was expected in the 1–6 s after the beep. This stressor included 26 beep sounds in total and lasted 5 min. The sounds had at least a window of 7 s between each other, with an average of 11.38 s (*SD* = 2.87). The presentation time of beep sounds were randomly generated beforehand in order to prevent the participant from recognizing a pattern in the sounds, which would make the listening task less stressful. The same sequence of sounds was used for each participant to make the experiment and the data comparable. After the 5-min period, the participants were asked to focus on their breathing for 2 min during the recovery period.

### Materials

The experiment was programmed with Python 2.7 and ran by Psychopy 1.8 (Peirce, 2009). Timestamps from the Python program were added to the physiological data (via the serial port and a voltage isolator) for experimental events. The Python program also wrote timestamps for these events to separate text files. The instructions were presented on a 15.4-in. Windows 7 laptop. A Philips SHP2000 headphone was used for the noise task.

#### Recording of physiological data

The physiological data was recorded with ProComp Infiniti System with BioGraph Infiniti Software-T7500M by Thought Technology and processed with MATLAB. To determine the validity of the skin conductance of the Empatica E4 wristband, the measurements were compared to the ones retrieved with the reference laboratory based apparatus on the fingers, which is a validated and recommended measurement of skin conductance (Dawson et al., 2007). The signal was retrieved from the intermediate phalanges (Edelberg, 1967). Some (Boucsein et al., 2012) recommend using the distal phalanges, however comparison within the present study showed no differences between the two phalanges. EDA was measured with the skin conductance sensor—SA9309M by Thought Technology (sampled at 256 Hz). Additionally, the ECG signal was recorded with two electrodes on the left and one electrode on the right wrist.

#### Characteristics of the wearable biosensor used

The biosensor used in the present study was the “E4 wristband” (Empatica, model E4, 2015). The wristbands of Empatica measure several different psychophysiological responses of the body (Garbarino, Lai, Tognetti, Picard, & Bender, 2015). In particular, the E4 wearable has four sensors: photoplethysmogram sensor, electrodermal activity sensor, three-axis accelerometer, and a temperature sensor. In this experiment only the data from the photoplethysmogram sensor (sampled at 64 Hz) and the electrodermal activity sensor (sampled at 4 Hz) were used. For uploading the data of the E4 wristband, the program Empatica Manager was used, which is a cloud-based program.

### Procedure

At the start of individual sessions, participants were asked to sit in front of the laptop. They were told that they would be attached to sensors during the experiment and to move as little as possible, because movement could contaminate retrieved data. Also, they were informed that all instructions would appear on screen and that the experiment would last approximately 45 min. Moreover, they were told that participation is voluntary, that they could stop the experiment whenever they wanted and that the data would be processed anonymously. After these instructions, participants were asked to read and sign an informed consent.

Next, the researcher attached the electrodes to the participants’ fingers and wrists. The E4 wristband was attached to the participants’ left wrist, the ECG to the right and left wrist, and the skin conductance sensors on the left ring finger and index finger. Additionally, the participants had to wear headphones during the experiment. After all sensors were attached, the researcher started the required programs needed. The researcher took a seat next to the participant, in order to heighten the stress received during the SSST, as this is primarily a social stressor. The instructions of the SSST and noise stressor appeared onscreen, respectively. Baseline and recovery period were given before and after each task. Furthermore, the participants were instructed to fill out a perceived stress scale (7-point Likert scale) after each task. These questionnaires were not further studied for the present article. After completion, the researcher removed the electrodes, debriefed the participants and thanked them for their participation.

### Time synchronization

A Python script created voltage changes in a dedicated channel of the amplifier at the onset of the various phases of the experiment and wrote the timestamps of these events to a log file. These timestamps could be combined with the timestamped E4 log files. The E4 manager software that was used to download the E4 log files and time synchronize the E4 wearable itself ran on the same computer that also ran the Python script. This ensured that the source of the timestamps for the different log files was identical.

### Data analysis

#### Data quality assessment

##### EDA

As recommended by Boucsein (2012), visual checks were performed on the skin conductance data to identify failed measurements (on both the RD and the E4 wearable): “nonresponding” (indicated by an absence of SCRs in a given measurement), and incorrect classification of SCRs. Two researchers examined the plot and their interrater reliability of nonresponders was determined. Of the 77 participants, 17 (22%) were specified as nonresponders and removed from the dataset. A final 60 participants were included in the research, which is more than the number required according to the power analysis. When either of the researchers classified the data as nonresponding, the complete data was removed (the interrater reliability was .78).

##### CVA

For the PPG signal, another approach was needed to assess the data quality. The PPG signal is less stable than the ECG signal, and the stability of the signal can fluctuate over time, meaning that not only some participants’ data should be completely removed, but also some parts of the signal could be too noisy to be interpretable. However, removing these partially noisy datasets entirely would lead to removing more data than necessary. Therefore, the data quality of the PPG signals was assessed throughout the signal with a signal quality index (SQI; Karlen, Kobayashi, Ansermino, & Dumont, 2012), on a scale from 0 to 100. On average, the signal quality per person was 72.9 (*SD* = 16.8). Experimental blocks, such as the baseline or stressor, that did not have consecutive data for at least 50% of the block, were deemed to have low SQI. For the baselines and the SSST, a SQI of at least 70 was needed, since these blocks are quite long (35 s). The data for the noise task needed to meet a higher quality level due to the short timeframes of relevant data, 1–6 s after each beep. In such a short interval (5 s), only four to six heartbeats would normally be registered—and even half of that small number for a consecutive signal when 50% of the data was available. Therefore, it is important that the heartbeats that are registered are correct, meaning that the data should have a high quality. This approach led to the removal of 45% of the data, and the removal of the complete dataset for seven participants, as their datasets did not have enough data quality. One participant was removed, since the HR data were biologically implausible (i.e., over 200 bpm). In the end, the data of 39 participants were included for further analysis. We will next discuss how comparisons will be made between a wearable and the RD at each of the three levels of our framework.

#### Signal comparison—Cross-correlation function

With a cross-correlation two signals are correlated with each other over time, and the autocorrelation with the prior time points in their own signal is modeled. Cross-correlation is therefore determined at the same time for both signals and at a number of intervals for which one of the signals is moved backward or forward in time. Each interval—which is called a *lag*—is proportional to the frequency of the signal. When a wearable measures, for example, at 4 Hz, one lag is 0.25 s. This method provides a measure of the coherence between two signals for each lag, taking into account the autocorrelation within the signals and a possible systematic delay between the signals. If a systematic delay is detected for all individuals (everyone’s cross-correlation is the highest at, e.g., lag 2), corrections for this delay could be executed.

The first two steps described in Box 2 make the two signals comparable without removing any relevant information. Boucsein (2012) describes that down sampling EDA data to more than 10 Hz does not result in significant improvement of the quality of the data. In the third step skin conductance response (SCR) is mentioned, which is the phenomenon of interest in EDA signals.

**Box 2** Steps to determine the cross correlation for EDA data.

**Table:** 

**1. Down and up sample the data to the same frequency.** The EDA signal of the RD data was down sampled to 16 Hz signal, since sampling higher than 16 Hz does not add to the data quality. The wearable data was up-sampled from 4Hz to 16 Hz, to make the sampling rate similar to the RD data. **2. Normalize and detrend the data.** Normalization was done to make two signals better comparable without losing viable information. Additionally, the data was detrended in order to make the data stationary, which is a prerequisite for a time series analysis like the cross correlation function. **3. Determine cross correlation at multiple time lags.** Time lag between -8 and +8 were considered, because a SRC can have a duration of multiple seconds. The sample frequency is 16 Hz meaning that a time lag of 16 represents one second and a time lag of 8 represents 0.5 s. **4. Find highest cross correlation with corresponding time-lag and plot these in a histogram.** To gain an overview of all found optimal cross correlations a histogram is made. From this is the most optimal cross correlation can be found for each participant.	

##### EDA

The cross correlation (see Box 2 for the required steps) is optimal for determining the similarity of the EDA signals, due to the autocorrelation displayed in the signal and the possible arrival delay of the signal. Such a delay might come from the difference in measurement location, since the E4 wristband measures at the wrist and the RD measures at the fingers. Hence, the travel distance between the wrist and the finger could be observable in the signal. The average length of a hand is 18 cm (Agnihotri, Purwar, Jeebun, & Agnihotri, 2005), which is more or less the distance between the fingers and the wrist and the transmission velocity is 1.2–1.4 m/s (Jänig, Sundlöf, & Wallin, 1983), therefore the max time lag between wrist and finger is 0.18 s. Which in the case of 16 Hz is a time lag of 4, or 0.25 s. However to create a safe window we propose a double window of lag 8, given that the previous numbers are based on physiological group averages. The presence of such a systematic delay can be determined and controlled for with a cross-correlation function.

##### CVA

No comparison can be made on the signal level between ECG and PPG signals, due to differences in the characteristics of the signals (see Fig. 2).

#### Parameter comparison—Bland–Altman plot

The Bland–Altman (1986) plot is a visual representation of the agreement between two devices on a particular parameter, not only for a person’s average (e.g., ICC), but also over the range of the parameter. The plot identifies how much the new method is likely to differ from the old instead of quantifying the actual agreement. If the wearable differs from the RD, possible patterns in the data could be further explored for structural biases. An overview of multiple biases to be detected in the data is given by Giavarina (2015). Even when agreement within certain limits exists between the RD and the wearable, smaller structural biases could still be present in the data (e.g., a significantly higher mean). Exploration of these biases is therefore always advised. The steps to arrive at the Bland–Altman plot are presented in Box 3.

Box 3 Steps to determine the Bland–Altman plot for EDA data.

**Table:** 

**Step 1.** Same as for the EDA cross correlation Step 1 (see Box 2). **2. Analyze the data.** Since this study aims to validate the wearable signals against a RD, the phasic activity coming from classical trough-to-peak analysis (TTP) was reported (threshold for an SCR amplitude was set at .01 μS) (Boucsein, 2012). The data were analyzed with Ledalab; therefore, the default settings for filtering and smoothing from the program were used (Benedek & Kaernbach, 2010). Note that different choices in filtering and smoothing can influence the results. **3. Retrieve the parameters from the timeframe determined**. Three parameters from the EDA data are evaluated with a Bland–Altman plot: Mean skin conductance level (SCL) The skin conductance level was based on the whole signal (start baseline – baseline after the noise task). The mean was calculated by averaging over the complete signal. Biological plausible values for SCL is between 0 and 16 μS (Braithwaite, Watson, Jones, & Rowe, 2013), the boundaries of the Bland–Altman plot are therefore ± 1.6 SC. **Number of SCRs** The SCRs in the signal were determined through trough to peak (TTP). The number of SCRs per minute was then determined. Biological plausible values for number of SCRs are on average 1–3 per minute according to (Braithwaite et al., 2013) and during high arousal 20-25 per minute (Boucsein, 2012), the boundaries of the Bland–Altman plot are therefore ± 2.5 SCRs. **SCRs total amplitude (S-AMPL)** The amplitude of a response was determined as the difference in conductance between response onset and response peak. The amplitudes were added in order to determine the total amplitude. The total amplitude is therefore a function of both the number of SCRs and the amplitude of all these SCRs. Biologically plausible values for amplitudes are between 0 and 3 μS and on average 0.30–1.30 μS according to (Braithwaite et al., 2013) and with 20–25 SCRs per minute the range of total amplitudes is between 0 and 0.3*20 = 6 μS when using the most conservative values. The boundaries of the Bland–Altman are therefore ± 0.6 μS. **4. Check for normality and missing data.** The assumption of the Bland–Altman is that the differences between the wearable and the RD are normally distributed. Therefore normality of the differences needs to be assessed visually. If the data appears not normal appropriate transformations (e.g. log transformations) can be used as suggested by Boucsein (2012). Additionally, the quantity of missing data can be viewed from these plots. If the amount of missing data is effecting the power, then inferences should be made with more caution or possibly no inferences can be made. **5. Create a Bland–Altman plot.** Plot the mean of the two measurements as the abscissa (*x*-axis) value, and the difference between the two values as the ordinate (*y*-axis) value. $\left(\frac{E4+ TT}{2},E4- TT\right)$ Additionally plot the two proposed boundaries and the 95% CI of the differences in a different color. Calculate the amount of data outside the CI, as follows: $\frac{count\left({\mu}_{\left(E4, TT\right)}-1.96{\sigma}_{\left(E4, TT\right)}>E4- TT>{\mu}_{\left(E4, TT\right)}+1.96{\sigma}_{\left(E4, TT\right)}\right)}{n}\ast 100$	

##### EDA

See Box 3.

##### CVA

See Box 4.

**Box 4** Steps to determine the Bland–Altman plot for CVA data.

**Table:** 

**1. Down- and up-sample the data to the same frequency.** Down-sample the RD data and up sample the wearable data from 64 Hz to a frequency of 200 Hz. **2. Analyze the filtered data** The raw ECG and PPG recorded was filtered with a combination of low-pass and high-pass filters between 5 and 15 Hz (Pan & Tompkins, 1985). For each segment of data, the peaks of normal R-waves were detected using a filter-bank-based algorithm developed by Pan and Tompkins. The peaks of the P-waves were detected by finding the local optima. The durations between successive peak locations were calculated to produce RR/PP intervals. The RR/PP intervals with a length less than 0.33 s or more than 1.5 s were deleted from time series. **3. Retrieve the parameters from the timeframe determined**. In Fig. 2, an example of an ECG and PPG signal is presented. In the ECG signal, the R peaks from the QRS complexes are illustrated. The distance between two successive R peaks is called the RR interval. From the PPG signal instead of the RR intervals, PP intervals are retrieved, which represent the time between the top of two peaks in the blood volume pulse signal. Next to the RR/PP intervals, a quality measure was determined, the signal quality index (SQI; Orphanidou et al., 2014). Parameters were only determined when at least 50% of the data had an SQI of 80%. Three parameters from the time domain were retrieved from the RR or PP interval data: **Mean RR/PP interval** The mean RR or PP interval is the mean over a period of time in which RR/PP intervals were retrieved. The mean PP interval is a surrogate measurement of the mean RR interval, and both can be converted into instantaneous HR. Biologically plausible values for instantaneous HR during a seated task are between 60 and 110 beats per minute (bpm), the boundaries of the Bland–Altman plot are therefore ± 5 bpm. **SD RR/PP Interval** The standard deviation over the RR or PP intervals. Since RR intervals are possible below 1 s the biologically plausible values for *SD* RR/PP interval are between 0 and 0.56 (O’Neal et al., 2016). The boundaries of the Bland–Altman are therefore respectively ± 0.06. **RMSSD** The root mean square of the successive differences is a time-domain measure which is related to HR variability. It can be calculated via the following formula: $\sqrt{\frac{1}{N-1}\left(\sum \limits_{i-1}^{N-1}{\left({\left(R-R\right)}_{i+1}-{\left(R-R\right)}_i\right)}^2\right)}$ Biologically plausible values for RMSSD are between 0 and max .71 s (O’Neal et al., 2016), the boundaries of the Bland–Altman are therefore ± 0.07 s. **Step 4 and 5.** Same as for EDA (see Steps 4 and 5 in Box 3).	

#### Event detection comparison—Event difference plots

The event difference plot is a visualization of the mean effect and its confidence interval in each task. Tasks are in this case a social stressor (SSST; Brouwer et al., 2018), and an environmental stimulus (noise task; Bali & Jaggi, 2015). The steps to arrive at the error plot are given below.

##### EDA

We expected a significant increase to the baseline for both the SSST and the noise task. For the SSST we expect that preparing to sing results in a significant difference to the baseline for total amplitude. We expected that the first few beeps in the noise task would lead to a higher total amplitude, but that this effect would habituate over beeps (see Box 5).

**Box 5** Steps to determine event difference plots for EDA data.

**Table:** 

**Step 1 and 2.** Same as for the EDA Bland–Altman plot (Box 3, Steps 1 and 2). **3. Retrieve an informative parameter for the variable of interest from the analyzed data**. Total amplitude was chosen as a stress parameter, since this is a combination of both the number of SCRs and the amplitude of the SCRs. **Step 4.** Same as for the Bland–Altman plot (see Step 4 in Box 4) **5. Make event difference plots of the data.** In order to visualize the effect and the agreement between the two devices, multiple plots are made. 1) A line plot with the mean and the *SE* per task with a line through the zero *y*-axis, where each individual is represented in a line. 2) A line plot with the differences between the wearable and the RD for each person are represented with a line, with the mean and the *SE* per task. Additionally plot the a priori defined boundary, which is the reference effect (difference between the stress task and baseline from the RD) of the variable of interest.	

##### CVA

For the RD we expect that preparing to sing results in a significant difference to the baseline for heart rate. For heart rate no assumptions on the habituation effect during the noise task are made a priori since this type of habituation effect is typically measured with EDA and not with HR (see Box 6).

Box 6 Steps to determine event difference plots for CVA data.

**Table:** 

**Steps 1 and 2.** Same as for the Bland–Altman plot CVA (see Box 4, Steps 1 and 2). **3. Retrieve an informative parameter for the variable of interest from the analyzed data**. Instantaneous HR was chosen as a stress parameter, since this is an often used measure (Schubert et al., 2009) and is for most people an intuitive measure related to CVA. **Steps 4 and 5.** Same as for the EDA parameter (see Box 5, Steps 4 and 5).	

## Results

This section describes the results of the validity assessment protocol on the E4 wearable for each of the three levels of our method.

### Signal comparison: Cross correlation function

#### EDA

**Fig. 5:**
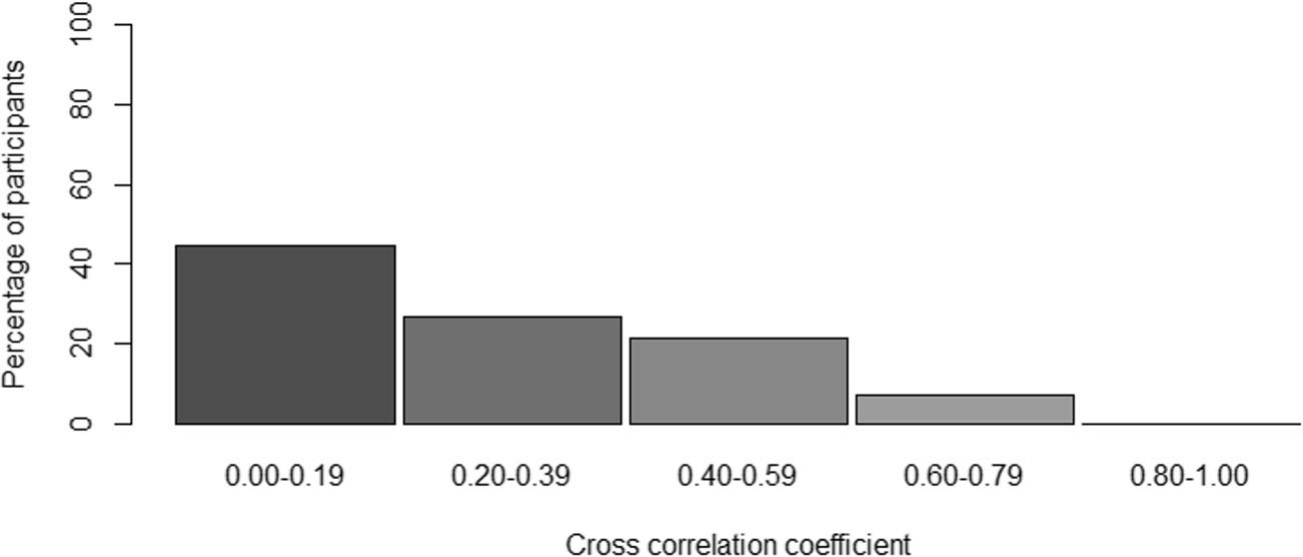
Histogram of the optimal cross-correlation found for a participant between – 8 and + 8 lags in time. The cross-correlation for each participant presented in the figure was determined on the basis of his or her most optimal lag

For the majority of participants, the correlation would be considered low (i.e., below .4); however, for some participants the correlation was .60–.79 (see Fig. 5). On average, the cross-correlation was .25. We found no evidence for a systematic time lag in the data, meaning that not all participants had the highest correlation at one specific lag, implying that the “optimal” lag differed per participant. It even differed between positive and negative lags.

**Fig. 6:**
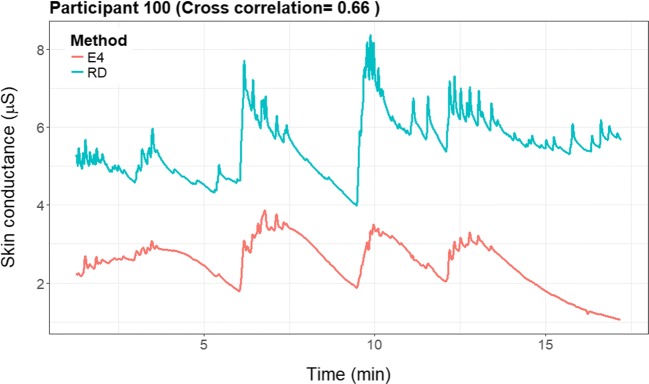
Cross-correlation plots of a participant with a high cross-correlation (.66)

**Fig. 7:**
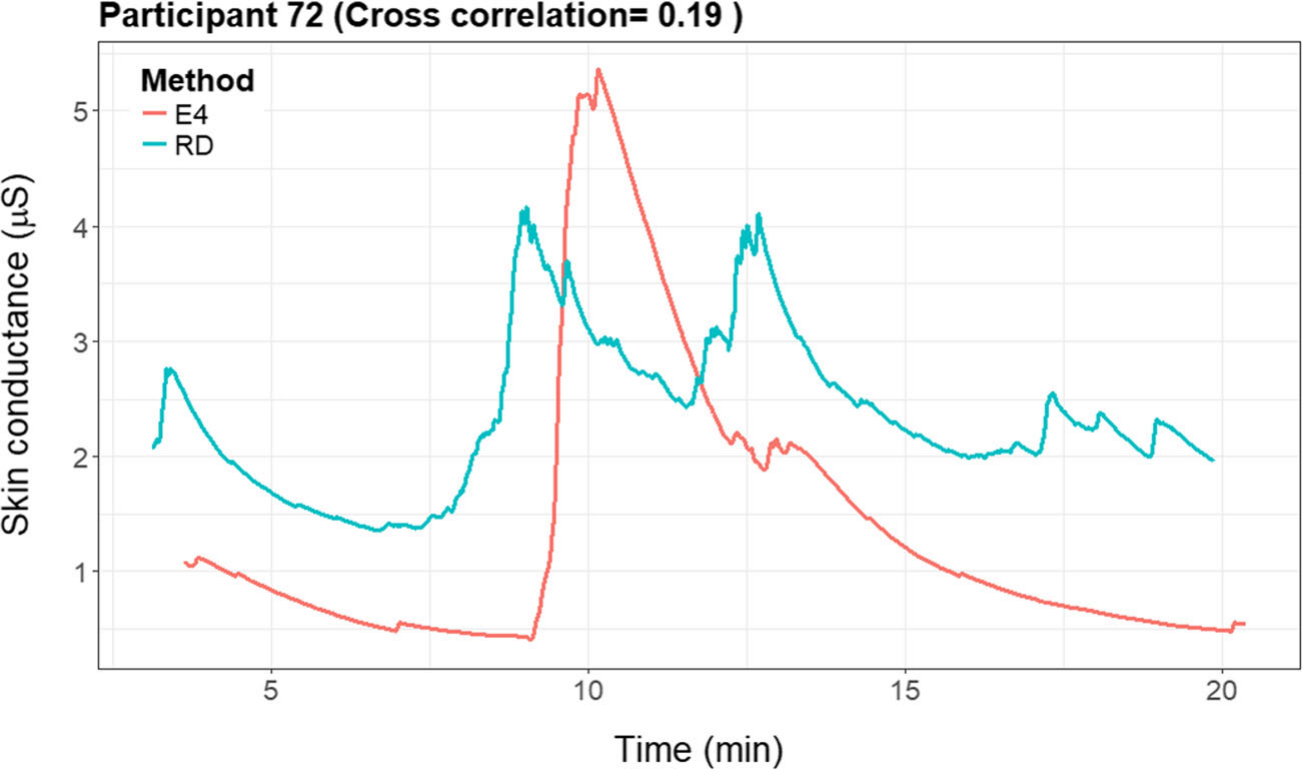
Cross-correlation plots of a participant with a low cross-correlation (.19)

For illustrative purposes, we provide the raw EDA signals of two representative participants, illustrating a prototypical high (see Fig. 6) and a low (see Fig. 7) cross-correlation. In the figure with the higher cross-correlation (participant 100: .66), one can see that the signals seem to change simultaneously.

Participant 72 (Fig. 7) had a very low cross-correlation (.19) in the signals. The E4 wearable showed one big and only a few smaller peaks in the data, whereas the RD shows two big peaks, one similar to the peak of the E4 wearable, and the other similar to a smaller peak in the E4 signal. There is some similarity between the signals; however, the amplitude and the timing of the peaks seem to differ.

### Parameter comparison: Bland–Altman plot

#### EDA

**Fig. 8:**
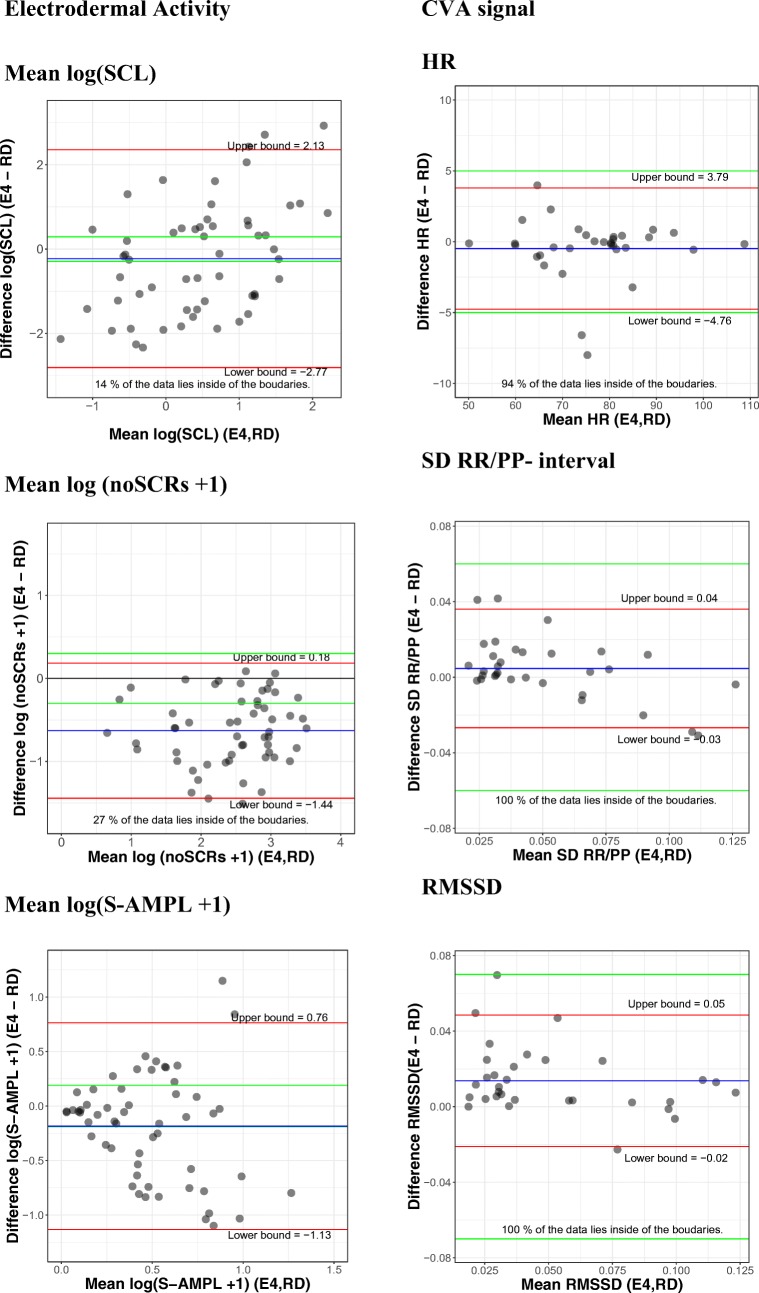
Bland–Altman plots for electrodermal activity, on the left, and cardiovascular activity (CVA), on the right. Each dot represents one participant. The difference between and the average of the two measures are represented on the *y*-axis and the *x*-axis, respectively. The green lines represent the a priori chosen acceptable boundaries, whereas the red lines (also marked with upper bound and lower bound) represent the actually found 95% confidence interval limits. At the bottom of each figure, the percentage of values within the proposed boundaries is given. HR, heart rate; RMSSD, root mean square of successive differences; RR/PP, the durations between successive RR (RD) or PP (E4) peaks; S-AMPL, amplitude of the skin conductance responses; SCL, skin conductance level; SCR, skin conductance responses

Bland–Altman plots for the paired measures of the mean SCL, total amplitude, and number of SCRs of the EDA signal are shown in Fig. 8. SCL, number of SCRs, and total amplitude are all transformed with a log transformation in order to achieve normality. The plots show that all parameters of the EDA lie outside the acceptable agreement. For the number of SCRs, a mean bias was found, since the mean difference lies below 0. The E4 wearable on average underestimated the number of SCRs. A reassessment with a lower threshold (0.001) for the E4 wearable did not improve the results.

This bias is not systematic, since a systematic bias would imply that there is always a two-SCR difference or that the difference increases linearly for a higher number of SCRs. However, more than one person has a number of SCRs above the zero line, meaning that the E4 wearable overestimated the SCRs for those participants. For total amplitude and SCL, the bias seems to increase when the amplitude increases. This implies that there was uncertainty among the complete range of amplitudes. Additionally, if the two large positive differences were removed from the SCL data, an underestimation by the E4 wearable would be found for both total amplitude and SCL.

#### CVA

Regarding the PPG/ECG analysis, the Bland–Altman plots of the mean, *SD*, and the RMSSD of the PP/RR interval can be found in Fig. 8. The plots show good agreement for all parameters between the RD and E4 wearable on *SD*. In all, 94% of the participant data and therefore the limits of agreement are between the ± 5-bpm boundaries for HR, 97% between the ± 0.06 boundary of the *SD* and 97% between the ± 0.07 boundary of the RMSSD.

### Event level: Event difference plots

To determine the validity of the E4 wearable, the ability to detect stress change was determined. Two tasks were used to determine stress reactions, namely a general social stressor, the SSST, and an event based environmental stressor, the noise task. For the EDA measurements all the data is complete. However, for the HR data 74% of the data is missing across the tasks of the SSST and only four persons have data for all four tasks. For the noise task 40% of the data is missing and two persons have complete data sets.

#### EDA

**Fig. 9:**
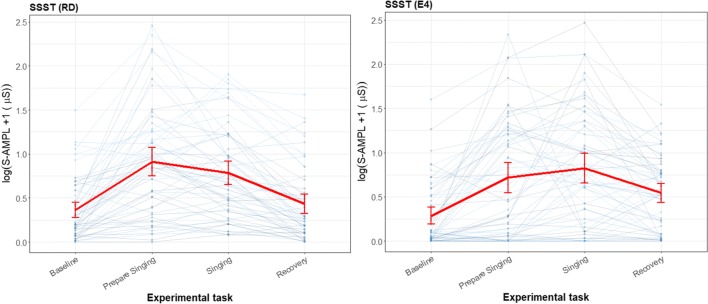
Line plot for the sing-a-song-stress task (SSST), measured with the reference device (RD) and the E4 wearable, with total amplitude of SCRs as the parameter of interest. Each thin line represents a participant, and the mean and its error bars are plotted in red. The *y*-scale is given as a square root, to show the difference in the lower regions and not have too much emphasis on the higher values. For the SSST, the first experimental task is a neutral baseline, the second is preparing to sing, the third is singing, and the last is recovery (another baseline) directly after singing

Figure 9 is a graphical representation of the baseline, preparing to sing, general stress response task (singing), and recovery period. The SSST shows the expected increase in total amplitude when preparing to sing (1.52 increase to the baseline) and while singing (0.98 increase to the baseline) for the RD. Similar effects are found for the E4 wearable; preparing to sing (1.13 increase to the baseline) and singing (1.39 increase to the baseline) resulted in very strong increases in the total amplitude of SCRs.

There was no significant difference between the E4 wearable and the RD in the baseline, preparing to sing, singing, and recovery baseline. The E4 wearable seems to have a longer recovery time than the RD (Fig. 9). The preparing-to-sing and singing tasks were both significantly different from the neutral baseline. This can be observed from the error bar plot, since the error bar intervals of the neutral task and the stress tasks did not overlap.

**Fig. 10:**
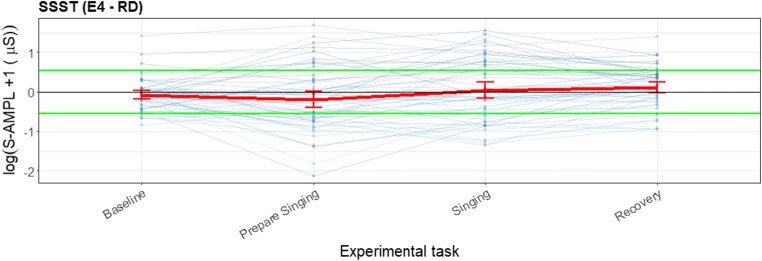
Error bar plot for the differences for each participant during the sing-a-song-stress task (SSST) and for the mean and standard error during each task (red line) for all participants, with total amplitude of SCRs as the parameter of interest (blue lines). The black line is the zero axis, and the green lines are the a priori defined boundaries (size of the reference effect). For the SSST, the first experimental task was a neutral baseline, the second was preparing to sing, the third was singing, and the last was the baseline directly after singing

As can be seen in Fig. 10, there was overlap between the error bars for all tasks related to the difference scores between the E4 wearable and the RD and the zero axis. Additionally, none of the error bars exceeded the a priori defined boundaries. For preparing to sing and singing, the error bars were larger than for the baseline.

**Fig. 11:**
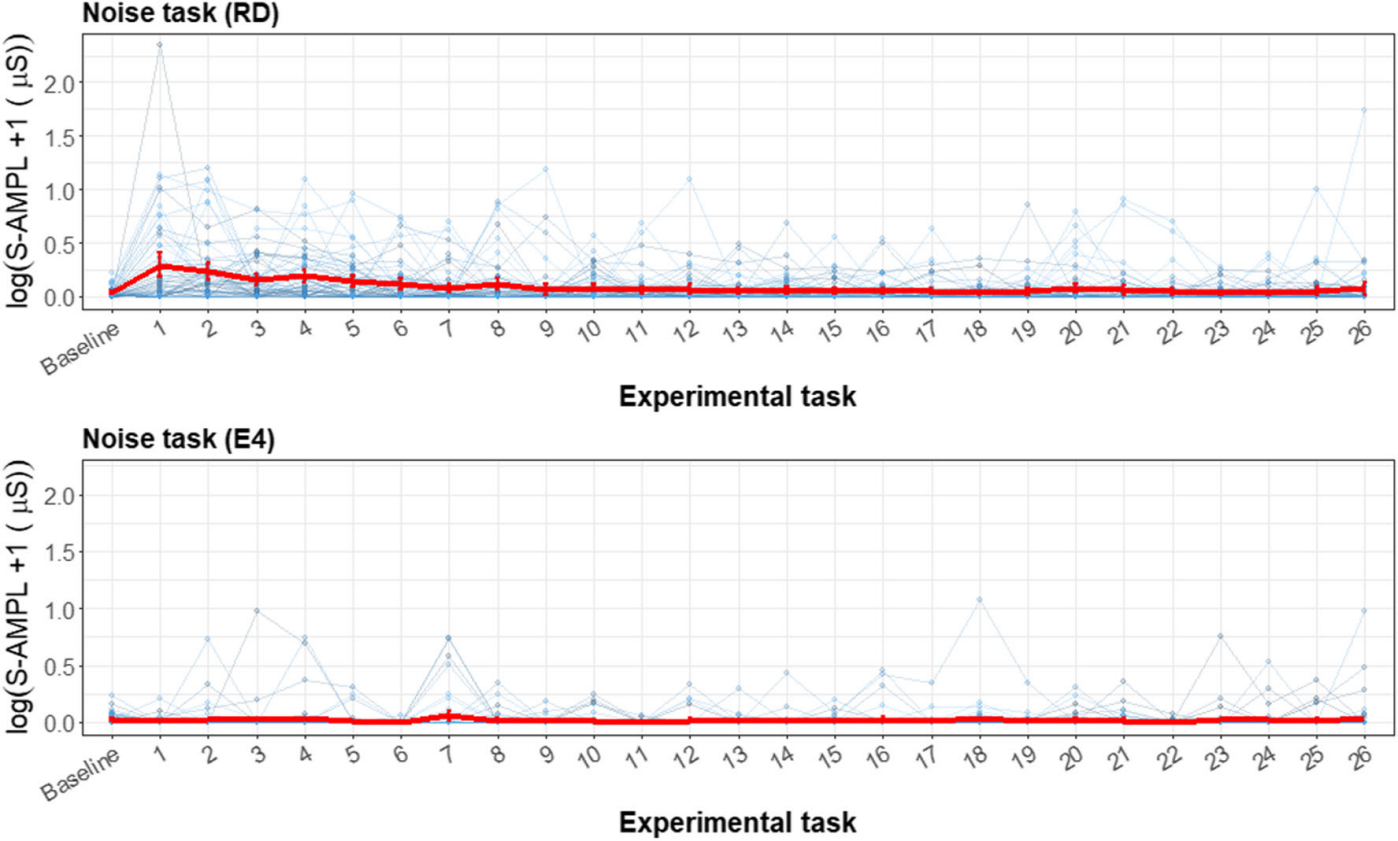
Error bar plots for each participant (thin blue lines) during the noise task, retrieved with the reference device (RD) and the E4 wearable. The mean and *SE* during each task for all participants, with the total amplitude of SCRs as the parameter of interest, are indicated with red lines. The *y*-scale is given as a logarithm, to show the difference in the lower regions and not have too much emphasis placed on the higher values. For the noise task, the same baseline is given as for the SSST, and every number represents a beep

In Fig. 11 the noise task is displayed, which consisted of a baseline and 26 loud beeps. For the event-habituation task, the RD worked as expected for the EDA signal. For the first few beeps the total amplitude was significantly higher, and after the first few this effect reduced, showing the expected physiological habituation effect. For the RD, there is a decrease in SCR amplitude of 4% with every successive noise stimulus. In contrast, the E4 wearable shows no significant increase from the baseline or decrease with every successive noise stimulus.

**Fig. 12:**
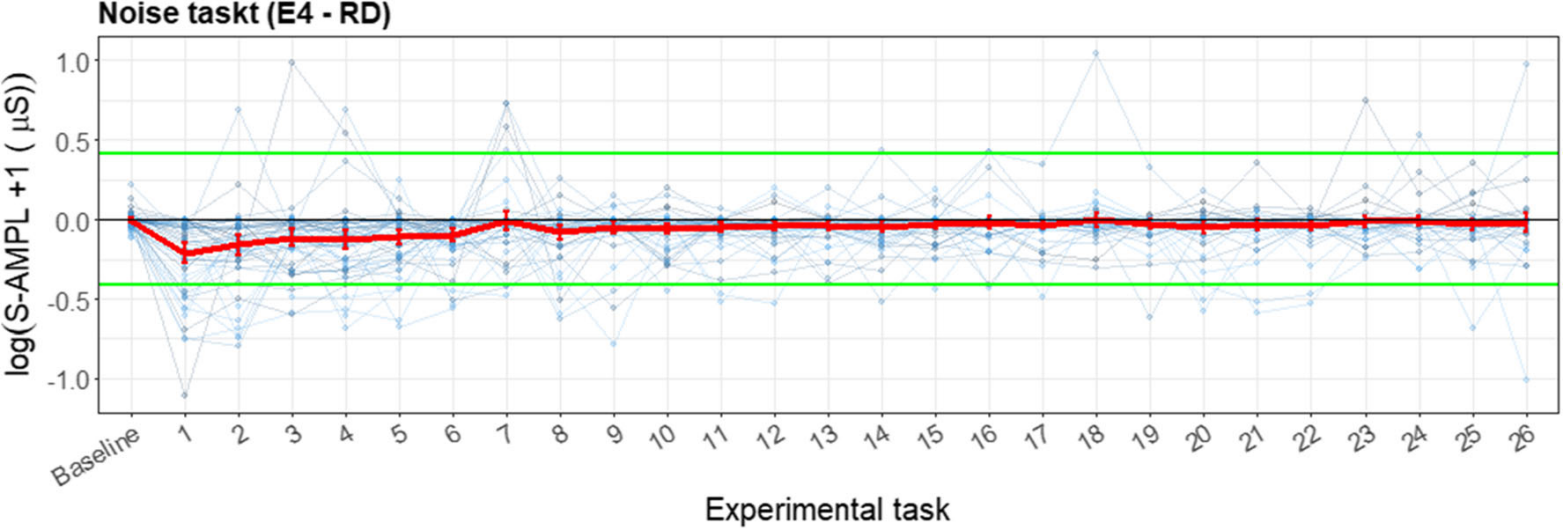
Error bar plot for the differences for each participant (blue lines) during the noise task. The means and *SE*s are also shown (red line) during each task for all participants, with the total amplitude of SCRs as the parameter of interest. The black line is the zero axis, and the green lines are the a priori defined boundaries (size of reference effect). The noise task has the same baseline given as for the SSST, and every number represents a beep

Even though the E4 wearable showed no significant effect for the noise task, a difference plot was made for illustrative purposes. In Fig. 12, the difference between the RD and the E4 wearable is visualized. For the baseline, the difference crossed the zero line. However, for the first few beeps the error bar lay outside the boundaries. For later beeps the differences became smaller, when the RD total amplitude values were closer to zero again.

#### CVA

**Fig. 13:**
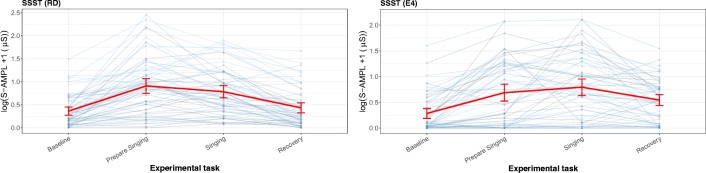
Error bar plot for each participant (blue lines) during the sing-a-song-stress task, retrieved with the reference device (RD) and the E4 wearable, with heart rate as parameter of interest. The overall means and SEs are also shown (red lines). The first experimental task was a neutral baseline; the second, preparing to sing; the third, singing; and the last, a baseline directly after singing

In Fig. 13, the event detection for both the RD and the E4 wearable are plotted for the SSST. For the SSST, a clear stress pattern is found.

**Fig. 14:**
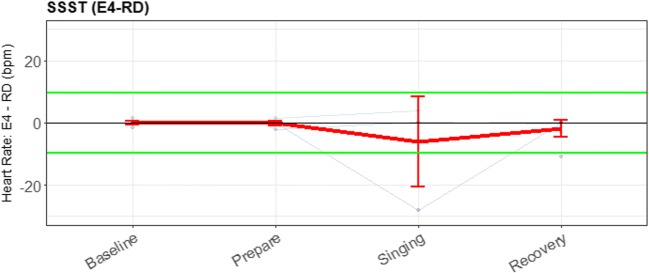
Error bar plot for the differences for each participant (blue lines) during the SSST. The means and *SE*s are also shown (red line) during each task for all participants, with heart rate as the parameter of interest. The black line is the zero axis, and the green lines are the a priori defined boundaries (size of the reference effect). For the SSST, the first experimental task was a neutral baseline; the second, preparing to sing; the third, singing; and the last, a baseline directly after singing

Figure 14 shows that when comparing the two devices, even less reliable data were available. The error bars are therefore not informative to determine the validity of the E4 wearable for these short, 30-s intervals.

For the SSST, no significant differences from the RD between the baseline and preparing to sing could be found. However we did observe a significant effect of singing (19.8 bpm, an increase of 25%). Note that during this third task (singing), breathing also might have heightened the HR. For the E4 wearable, no significant changes in HR could be found for preparing to sing or singing. Additionally, from the lack of lines in the plot, it is clear that not enough data of acceptable quality were present to perform this analysis. Figure 14 was again made for illustrative purposes, since the lack of data makes a meaningful comparison impossible.

**Fig. 15:**
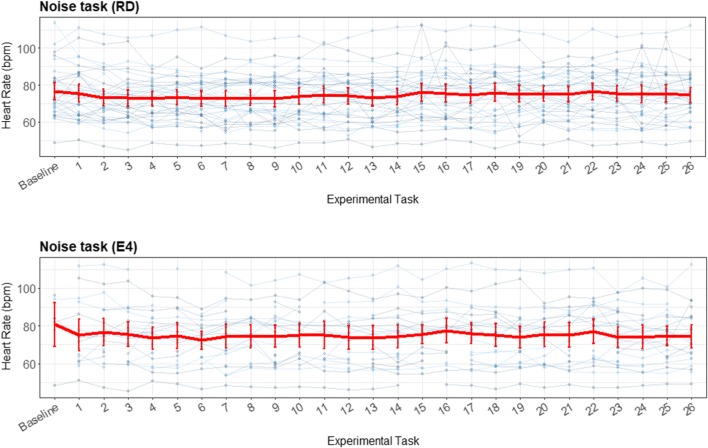
Error bar plot for each participant (blue lines) during the noise task, retrieved with the reference device (RD) and the E4 wearable. The overall means and *SE*s are also shown (red lines), displaying little variation

**Fig. 16:**
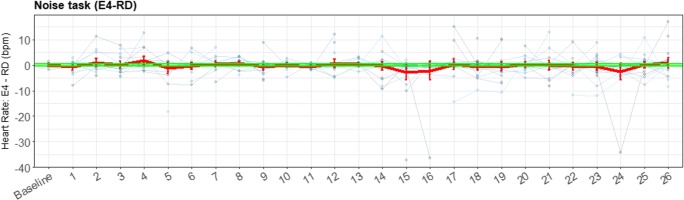
Error bar plot for the differences for each participant (blue lines) during the noise task. The means and *SE*s are also shown (red line) for all participants, with heart rate as the parameter of interest. The black line is the zero axis, and the green lines are the a priori defined boundaries (size of the reference effect, here very close to zero). The noise task has the same baseline given as for the SSST, and every number represents a beep

Figure 15 shows an almost random pattern for the noise task. It seems that the RD had a higher HR for the first beep than for subsequent beeps; however, this was not a significant difference, and could therefore have been random. The beeps of the E4 wearable also did not differ significantly. The task does not impute enough cardiovascular reaction to the environmental stressor, and therefore no comparison could be made for this stress task between the E4 wearable and the RD. The visualization of the difference between the RD and the E4 wearable in Fig. 16 is presented to illustrate (so we can later discuss) the importance and meaning of the various elements of the presented plots.

Figure 16 shows boundaries very close to the zero line, since the effect on HR of the stress task was small. Therefore, almost all error bars lie outside these boundaries. However, in this plot again the lack of data can be seen, and therefore no actual inferences—besides the lack of data of acceptable quality produced by the E4 wearable at these short timeframes—can be drawn from this plot.

## Discussion

This study was a first attempt to standardize the validity assessment of wearable technology measuring physiological signals. The proposed validity assessment protocol makes it possible to make clearer inferences, due to the advancement of explicit decision criteria upfront as recommended by Giavarina (2015). The clear decision criteria in combination with standardized methods lead to easier and clearer comparison between validation studies. In earlier studies, a variety of statistical methods (Zaki et al., 2012) was used to analyze validity on different unspecified levels and researchers often made inferences about the validity while having to specify their own criteria (Kottner et al., 2011), often without specifying them a priori. This led to ambiguous inferences about the wearable under evaluation. For example, Payne et al. (2016) and van Dooren et al. (2012) conclude that the RD is always better, but the wearable might be usable in an ambulatory situation. With the protocol presented here, we show that the E4 wearable is valid for heart rate, RMSSD and SD at the parameter and detection level, and for total amplitude of skin conductance responses for the detection level when studying strong sustained stressors. This conclusion is better supported by empirical evidence, yet also more nuanced than previous research. Below we will first discuss the method and its strengths and limitations after which we will discuss the results regarding the E4 wearable. We will show that the results regarding the E4 wearable are mainly similar to and extensions of previous findings, but are now provided within a strong framework to synthesize all the findings and to arrive at firm conclusions.

### Validity assessment protocol

The novelty of the proposed protocol lies in the explicit determination of the three levels, the choice of proper statistical analysis and the definition of decision criteria upfront. The protocol we designed is therefore not a completely novel protocol, but rather a combination of multiple existing statistical analyses (Bland & Altman, 1986; Brandt & Williams, 2007), diminishing the need for thorough validation of the analyses themselves. Zaki et al. (2012) argued that there are numerous improper methods to analyze validity. The choice of statistical analysis is therefore certainly not arbitrary and often turns out being incorrectly applied (Kottner et al., 2011). Researchers often determine the use of the statistical analysis without consulting a statistical expert (Zaki et al., 2012). The aim of this study was to suggest statistical visualizations that minimizes the chance of being applied incorrectly. For example instead of a repeated measures ANOVA, in which an *F* statistic and its corresponding *p* value need to be assessed, we proposed an event difference plot, in which overlapping error bars need to be assessed. In these visualizations missing data and nonnormality are visible and not hidden. The statistical analyses used in this study are already established, and above all, proper ways to analyze the validity at each level (Zaki et al., 2012). Since the statistical analyses are already established, the validity of the E4 wearable could be assessed to demonstrate the protocol proposed. The presented study is therefore an important step in the standardization and therefore especially the improvement of the statistical quality of validity studies. Additionally, we recommended to explicitly investigate and report on three levels of analysis, making validity studies thorough, transparent, and comparable. Researchers already often analyze validity on one or multiple levels (Ollander et al., 2016; Payne et al., 2016) of the ones proposed here, without explicitly specifying the levels of analysis. It could be argued that the three levels of analysis are interdependent, meaning that if agreement exists on one level, this will also apply to the other levels. This is only partly true, though. Excellent validity at signal level will always ensure validity at both the parameter and event level analyses. Conversely, lower signal validity does not directly imply invalidity at the parameter or event level, since the source of the low signal validity is unclear. For example the ECG and PPG are different signals that will lead to low signal validity, which does not immediately mean that HR cannot be correctly determined by both measures. This is an important notion in the case of wearables, in which a high-signal-level concordance with RD is only a theoretical possibility. In the future, a gold standard for wearable devices might become available, making high signal concordance realistic. Consequently, not differentiating between levels of analysis could lead to mistakenly disqualifying wearables when merely signal-level validation is assessed, whereas often event-level analyses are targeted in studies using the wearable. In this study the E4 wearable is indeed found to have low signal validity, yet is still usable when targeting certain parameters (HR, RMSSD, and *SD*). However, at event level with typical short time scales these variables show to have a lot of data loss. We argue that such wearables without sufficient signal validity could still be of use when thoroughly investigating the average person parameters for longer periods of time.

Some of the decision criteria we propose are well-known. For the event level, the decision criterion defined is already established; in an error bar plot it is customary to look at significance. For the cross-correlation (parameter level), Evans (1996) provided the categorization of the correlation coefficients. We argued that the cross-correlation should be very high for all participants. The main reason is that when a lower cross-correlation is found, it is often not possible to determine the systematical biases causing this lower correlation and therefore it is impossible to adjust for this bias. We assume that two gold-standard measurements would result in a very high (> .8) cross-correlation between two signals, were differences would only results from small measurement error cause by for example small variations in electrode placement. However, it would be preferable to evaluate this in future research with the present validity assessment protocol. For the parameter level, the decision criteria for the Bland–Altman plot are almost always missing (Giavarina, 2015), making a decision about the validity impossible. Giavarina advises to define such criteria upfront. However, at present these criteria were not defined before, except for HR (AAMI, 2003), meaning that the criteria in this study are the first to judge validity on the basis of a Bland–Altman plot. Again, we decided on quite stringent criteria, that were the elaboration on the criteria available for HR (AAMI, 2003). Finally, for the event difference plots we decided on a boundary resembling the “true” effect measured by the RD. This is the largest boundary acceptable in this case and if the device does not fit, then we have to accept that it is not valid. Obviously, there can be discussion about these criteria, but we have laid the groundwork to now enable this discussion. Future research should focus on repeating and checking the findings on this and multiple other wearables, in order to test and optimize these criteria.

This study has the limitation of not being situated in an ambulatory setting, for which wearables are often used (Giles et al., 2016; Majumder, Mondal, & Deen, 2017). The problem with assessing the validity of the E4 in ambulatory settings is that the conventional devices are not wireless, making the signal coming from the ‘gold standard’ unusable due to motion artifacts. There are, however, alternatives to the “gold standard” (e.g., the VU-AMS) that have been shown to be valid alternatives (for CVA measures) and that can be applied in an ambulatory context (Willemsen, De Geus, Klaver, Van Doornen, & Carroll, 1996). Additionally, the wearable works best in a seated setting, since activity leads to more artifacts in the data. We found 45% of the PPG and 22% of the EDA data to be unusable. Zheng and Poon (2016) found 78% EDA artifacts in a single case study in an ambulatory setting. A controlled setting was therefore chosen, since performing an experiment in an ambulatory setting will increase the amount of data loss, therefore increasing the number of participants needed in order to obtain the same statistical power. The use of the controlled setting is not expected to influence the validity of the data, since the stressors presented were chosen similarly to situations experienced in daily life (Brouwer & Hogervorst, 2014). Given that the protocol is now developed a new study could be done focusing on applying it for an ambulatory setting with longitudinal measurements.

### Application to E4 wearable

#### Signal level—EDA/CVA

The E4 wearable should not be used when wanting to retrieve the exact same signal to the signal retrieved from the RD. The cross-correlation coefficient for the EDA signals were lower than .80 for all participants, whereas a cross-correlation higher than .80 for all participants was determined as the criterion for validity on the signal level. These findings are in line with Ollander et al. (2016), who found no visual resemblances between the two signals. For CVA, these coefficients could not be determined due to use of a different measurement technique. As we argued before, in the case of wearable technology, the signals are not expected to have high cross correlation due to placement differences and usage of alternative techniques. This does not necessarily imply invalidity at both higher aggregate levels, though. Therefore, additional tests at the parameter and the event level, to test for validity, were performed.

#### Parameter level—EDA

At the parameter level, the E4 wearable is found not to be usable for EDA parameters. Both Payne et al. (2016) and van Dooren et al. (2012) expect that the wrist site is less responsive for EDA, leading to an underestimation of the E4 on total amplitude, number of SCR’s and mean SC level. This is in line with the findings for these parameters. If targeted effects are evaluated that are much stronger than a 10% increase, a parameter might still be usable as shown for total amplitude in the event level of this study. For example if a stressor is expected to increase total amplitude by 40%, then a measure being off by 10% at the parameter level is less problematic than when a stressor is expected to increase the total amplitude by 5%.

#### Parameter level—CVA

The CVA parameter results showed that heart rate can be validly determined within the boundaries by the E4 wearable as evidenced by the pilots of Ollander, Godin, Campagne, and Charbonnier (2016); McCarthy, Pradhan, Redpath, and Adler (2016); and Zheng and Poon (2016). However, the *SD*s of the differences for instantaneous HR are larger than the max difference SD given the power, meaning that there is more than a 10% likelihood that this agreement would not be found again when this study is replicated. Therefore replication is needed, advisable with a larger sample (*n* = 347). The other CVA parameters tested, namely RMSSD and *SD* HR can validly be retrieved from the E4 wearable when compared to the RD with 90% power. This is in line with the findings of Ollander et al. (2016) who reported that next to mean heart rate also *SD* HR could be determined acceptably by the E4 wearable. Zheng and Poon and McCarthy et al. did not determine parameters besides HR, and Ollander et al. only had a small sample as foundation, therefore no strong inferences about these parameters can be drawn.

#### Event level—EDA

The E4 wearable can be used to retrieve stress responses to a short sustained high social stressor, in this case the SSST (Brouwer et al., 2018). The E4 wearable detected stress responses in the SC signals that could be differentiated from the baseline. In contrast, the E4 did not detect the physiological habituation effect (which was clearly present for the RD) to a set of repeated sounds. These findings are in line with Ollander et al. (2016), Payne et al. (2016), and van Dooren et al. (2012), who also found or expect that the wrist is only sensitive to larger stressors. However, during singing and the after singing baseline, the wearable gave a significantly higher estimate than the conventional measure indicating invalidity. Multiple causes could be identified to cause this discrepancy. A plausible explanation could be that singing activates more thermoregulation, which is differently tracked by the E4 wearable (e.g., the larger surface of the wearable than the finger causes slower heat dissipation; Boucsein, 2012) than by the RD. Therefore, if a researcher is interested in the “cool down” process, the wearable might be invalid for this type of process. However, this study was not designed to investigate “cool down” effects and therefore no definite conclusions can be drawn.

#### Event level—CVA

For CVA, the results are inconclusive, since the SSST stressor was not detected by the RD and there was too much data loss, while measuring with the E4 wearable. The noise task did show similar results: the responses measured with the RD could not be distinguished from each other. This shows that the SSST and the noise task are not a strong enough stressor to elevate heart rate, which is in contrast to the findings of Brouwer and Hogervorst (2014) but in line with the findings of Alvarsson, Wiens, and Nilsson (2010). Additionally, there is too much data loss from the E4 wearable. This might be caused by the duration of the stressors, since the parameters determined over the whole data set showed less data loss. Therefore, no inferences can be drawn about small stress responses measured with heart rate. A different and possibly longer stressor is needed to elevate heart rate. We recommend to update the protocol and include a combination of multiple longer lasting stressors as implemented by for example (Reinhardt, Schmahl, Wüst, & Bohus, 2012) or multiple replications of the present stressors to improve the data quality.

### Further research

The presented validity assessment protocol showed results for the E4 wearable that are in line with the (limited) findings in the prior literature. We therefore think we provided a statistically sound, comprehensive and fast replicable framework for both researchers and clinicians. Nevertheless, additional research on the E4 wearable to verify the results is needed, since the validity of the E4 wearable in prior research was only piloted (e.g., McCarthy et al., 2016; Ollander et al., 2016). Additionally, the protocol could be further established by applying it to assess multiple other wearables, different populations, ambulatory settings with limited motion, other constructs of interest and nonlinear classification, since it currently only permits inferences for linear mean detection.

### Conclusion

This study has proposed a validity assessment that arose from the need for a systematic and comprehensive, yet easy replicable, validity assessment protocol, given the many wearable devices already used in scientific studies and clinical practice. The strengths of the protocol lie in the use of multiple levels of analysis, the clear decision framework, and the prerequisites of fast-to-replicate, statistically sound analyses that leave little room for incorrect interpretations. This protocol provides a framework for more comparable validation studies of physiological signals from new wearable technology and allows for a well-argued choice to use such a wearable, given the nature of the research. Given the growing presence of these new technologies in scientific, everyday, and also medical contexts, an objective and well-substantiated judgment about the quality of a particular wearable is important.

As for the E4, in ambulatory assessment the wearable should only be used when trying to explore physiological effects due to larger stressors and when determining a person’s average HR, *SD*, or RMSSD over a longer period of time. Sudden, short-lived stressors, such as being startled by the ringing of the phone, or possible habituation effects as a result of exposure to repeated information cannot be validly detected. We argue that physiological changes during a workday can be tracked by the E4 wearable against major, sustained stressors (e.g., a challenging team meeting) that are marked during the day.

## Appendix: Detailed power analysis

### Signal level—Cross-correlation

As we mentioned before, because only a correlation of .8 would be seen as acceptable, this would lead to an effect size of 1.8 [.8^2^/(1 – .8^2^)] and a sample size of 6, which is unrealistically low for the other analyses. This power analysis was performed using G*Power, version 3.1.9.2 (Faul, Erdfelder, Buchner, & Lang, 2009). Bonett (2002) proposed to investigate the confidence interval (CI) of an ICC instead of comparing it to a constant, leading to a sample size of 55 when the correlation is .8 and the width of the CI is .2. We recommend extending this calculation to the cross-correlation, since these measures are comparable when there is no mean shift (which can be corrected for when present). Furthermore, when there is a difference in variance between the ICC and the cross-correlation (McGraw & Wong, 1996), the ICC is more stringent than the cross-correlation.

### Parameter level—Bland–Altman plot

The appropriate power for the Bland–Altman analysis is dependent on the expected mean difference, the expected standard deviation of these differences, and the proposed boundaries. Limited prior research has been performed on validity assessment of the E4 or comparable devices with Bland–Altman plots; therefore, we cannot base the values on extensive prior research. However, Matsumura and Yamakoshi (2013) did assess the validity of a comparable PPG sensor with a Bland–Altman plot and found a mean difference of 0.20 bpm with an *SD* (of this difference) of 0.63 bpm for HR. This would result in an appropriate suggested sample size of six participants for a power of .90. This is probably an optimistic situation that cannot be transferred to all CVA and EDA parameters.

Therefore, we assessed the consequences of using a sample of 55, as was proposed for the cross-correlation. This resulted in a 1.75 *SD* of the differences that could be detected with .90 power for a mean difference of 0. We used a mean difference of 0, since we could correct for the mean differences according to Giavarina (2015), and were therefore only interested in the *SD* of these differences. In Table 1 all maximum difference *SD*s for the proposed parameters can be found, given the boundaries determined later in the Method section. In this power analysis, the mean difference was set to 0 and the power was 90%. Statistical analyses were performed using MedCalc for Windows, version 18.11.3 (MedCalc Software, Ostend, Belgium). Note that when the *SD*s found for the E4 exceeded this *SD*, the Type 2 error would increase and there would be less confidence in the conclusions, so that more research should be performed.

**Table 1:** Different measures with the max *SD* difference given the boundary determined below and a sample size of earlier analysis

Measure	Max *SD* Difference (90% Power)	Boundary (10% of Plausible range)	Sample size
SCL	0.56 μS	1.6 μS	53
NoSCRs	0.88/min	2.5/min	55
TAmp	0.21 μS	0.6 μS	53
meanHR	1.75 bpm	5 bpm	53
sdHR	0.021 s	0.06 s	47
rmssdHR	0.024 s	0.07 s	53

The sample size determined by for the specific power analysis is given in the last table

### Event level—Event difference plots

For the event level, two devices and 56 persons at four measurement moments gives .90 power to detect an effect size *f* of .25 or larger, which is a moderate effect. This can be transformed to a Cohen’s *d* of 0.5 (Lenhard & Lenhard, 2014). For HR, Brouwer and Hogervorst (2014) found an increase of 15.3 bpm (Cohen’s *d*: 1.63), and for EDA they found an increase of 10.9 μS (Cohen’s *d*: 0.81). For a noise task, Alvarsson, Wiens, and Nilsson (2010) found an effect of *t*(39) = 15.66 for EDA (Cohen’s *d*: 5.02). Both the effect sizes are large enough to detect with the power that is provided by a sample of 55 persons.

## References

[CR1] Agnihotri A, Purwar B, Jeebun N, Agnihotri S (2005). Determination of sex by hand dimensions. Internet Journal of Forensic Science.

[CR2] Alvarsson JJ, Wiens S, Nilsson ME (2010). Stress recovery during exposure to nature sound and environmental noise. International Journal of Environmental Research and Public Health.

[CR3] Association for the Advancement of Medical Instrumentation. (2002). Cardiac monitors, heart rate meters, and alarms. American National Standard (ANSI/AAMI EC13: 2002) Arlington, VA, 1-87.

[CR4] Association for the Advancement of Medical Instrumentation. (2002). Cardiac monitors, heart rate meters, and alarms. American National Standard (ANSI/AAMI EC13: 2002) Arlington, VA, 1-87.

[CR5] Bali A, Jaggi AS (2015). Preclinical experimental stress studies: Protocols, assessment and comparison. European Journal of Pharmacology.

[CR6] Benedek, M., & Kaernbach, C. (2010). A continuous measure of phasic electrodermal activity. *Journal of Neuroscience Methods*, *190*(1), 80–91. 10.1016/j.jneumeth.2010.04.028 10.1016/j.jneumeth.2010.04.028 PMC2892750 20451556

[CR7] Berntson GG, Quigley KS, Lozano D, Cacioppo JT, Tassinary LG, Berntson GG (2007). Cardiovascular psychophysiology. *Handbook of psychophysiology*.

[CR8] Bland JM, Altman D (1986). Statistical methods for assessing agreement between two methods of clinical measurement. Lancet.

[CR9] Bonett DG (2002). Sample size requirements for estimating intraclass correlations with desired precision. Statistics in Medicine.

[CR10] Boucsein W (2012). *Electrodermal activity*.

[CR11] Boucsein W, Fowles DC, Grimnes S, Ben-Shakhar G, Roth WT, Dawson ME, Filion DL (2012). Publication recommendations for electrodermal measurements. Psychophysiology.

[CR12] Braithwaite JJ, Watson DG, Jones R, Rowe M (2013). A guide for analysing electrodermal activity (EDA) & skin conductance responses (SCRs) for psychological experiments. Psychophysiology.

[CR13] Brandt PT, Williams JT (2007). *Multiple time series models*.

[CR14] Brouwer A-M, Hogervorst MA (2014). A new paradigm to induce mental stress: The Sing-a-Song Stress Test (SSST). Frontiers in Neuroscience.

[CR15] Brouwer A-M, van Beurden M, Nijboer L, Derikx L, Binsch O, Gjaltema C, Noordzij M, Ham J, Spagnolli A, Blankertz B, Gamberini L, Jacucci G (2018). A comparison of different electrodermal variables in response to an acute social stressor. *Symbiotic interaction*.

[CR16] Dawson ME, Schell AM, Filion DL, Cacioppo JT, Tassinary LG, Berntson GG (2007). The electrodermal system. *The handbook of psychophysiology*.

[CR17] Edelberg, R. (1967). Electrical properties of skin. In C. C. Brown (Ed.), *Methods in Psychophysiology,* 1–53. Baltimore: Williams & Wilkins.

[CR18] Egilmez, B., Poyraz, E., Zhou, W., Memik, G., Dinda, P., & Alshurafa, N. (2017). UStress: Understanding college student subjective stress using wrist-based passive sensing. In *2017 IEEE International Conference on Pervasive Computing and Communications Workshops (PerCom Workshops)* (pp. 673–678). Piscataway, NJ: IEEE Press. 10.1109/PERCOMW.2017.7917644

[CR19] Evans JD (1996). *Straightforward statistics for the behavioral sciences*.

[CR20] Faul F, Erdfelder E, Buchner A, Lang A-G (2009). Statistical power analyses using G*Power 3.1: Tests for correlation and regression analyses. Behavior Research Methods.

[CR21] Garbarino, M., Lai, M., Tognetti, S, Picard, R., & Bender, D. (2015). Empatica E3—A wearable wireless multi-sensor device for real-time computerized biofeedback and data acquisition. In *Proceedings of the 2014 4th International Conference on Wireless Mobile Communication and Healthcare—“Transforming Healthcare Through Innovations in Mobile and Wireless Technologies,” MOBIHEALTH 2014* (pp. 39–42). Piscataway, NJ: IEEE Press. 10.4108/icst.mobihealth.2014.257418

[CR22] Giavarina D (2015). Understanding Bland–Altman analysis. Biochemia Medica.

[CR23] Giles D, Draper N, Neil W (2016). Validity of the Polar V800 heart rate monitor to measure RR intervals at rest. European Journal of Applied Physiology.

[CR24] Jänig W, Sundlöf G, Wallin BG (1983). Discharge patterns of sympathetic neurons supplying skeletal muscle and skin in man and cat. Journal of the Autonomic Nervous System.

[CR25] Jennings JR, Gianaros PJ, Cacioppo J, Tassinary LG, Berntson GG (2007). Methodology. *Handbook of psychophysiology*.

[CR26] Karlen W, Kobayashi K, Ansermino JM, Dumont GA (2012). Photoplethysmogram signal quality estimation using repeated Gaussian filters and cross-correlation. Physiological Measurement.

[CR27] Kayhan, V. O., Chen, Z. (Chris), French, K. A., Allen, T. D., Salomon, K., & Watkins, A. (2018). How honest are the signals? A protocol for validating wearable sensors. Behavior Research Methods, 50(1), 57–83. 10.3758/s13428-017-1005-4 10.3758/s13428-017-1005-4 29330762

[CR28] Kirschbaum, C., Pirke, K.-M., & Hellhammer, D. H. (1993). The ‘Trier Social Stress Test’--a tool for investigating psychobiological stress responses in a laboratory setting. Neuropsychobiology, 28(1–2), 76–81. 10.1159/000119004 8255414

[CR29] Kottner, J., Audige, L., Brorson, S., Donner, A., Gajewski, B. J., Roberts, C., . . . Streiner, D. L. (2011). Guidelines for reporting reliability and agreement studies (GRRAS) were proposed. *International Journal of Nursing Studies, 48,* 661–671. 10.1016/j.ijnurstu.2011.01.016 10.1016/j.ijnurstu.2011.01.016 21514934

[CR30] Lenhard, W., & Lenhard, A. (2014). *Computation of effect sizes* (Unpublished report). 10.13140/RG.2.1.3478.4245

[CR31] Majumder S, Mondal T, Deen MJ (2017). Wearable sensors for remote health monitoring. Sensors.

[CR32] Matsumura K, Yamakoshi T (2013). iPhysioMeter: A new approach for measuring heart rate and normalized pulse volume using only a smartphone. Behavior Research Methods.

[CR33] McCarthy, C., Pradhan, N., Redpath, C., & Adler, A. (2016). Validation of the Empatica E4 wristband. *2016 IEEE EMBS International Student Conference: Expanding the Boundaries of Biomedical Engineering and Healthcare, ISC 2016—Proceedings*, (pp. 4–7). Piscataway, NJ: IEEE Press. 10.1109/EMBSISC.2016.7508621

[CR34] McCleary R, Hay RA, Meidinger EE, McDowall D (1980). *Applied time series analysis for the social sciences*.

[CR35] McGraw KO, Wong SP (1996). Forming inferences about some intraclass correlation coefficients. Psychological Methods.

[CR36] Nunan D, Nunan D, Jakovljevic DG, Donovan G, Hodges LD, Sandercock GRH, Brodie DA (2008). Levels of agreement for RR intervals and short-term heart rate variability obtained from the Polar S810 and an alternative system. European Journal of Applied Physiology.

[CR37] O’Neal WT, Chen LY, Nazarian S, Soliman EZ (2016). Reference ranges for short-term heart rate variability measures in individuals free of cardiovascular disease: The Multi-Ethnic Study of Atherosclerosis (MESA). Journal of Electrocardiology.

[CR38] Ollander, S., Godin, C., Campagne, A., & Charbonnier, S. (2016). A comparison of wearable and stationary sensors for stress detection. In *2016 IEEE International Conference on Systems, Man, and Cybernetics* (pp. 4362–4366). Piscataway, NJ: IEEE Press. 10.1109/SMC.2016.7844917

[CR39] Orphanidou C, Bonnici T, Charlton P, Clifton D, Vallance D, Tarassenko L (2014). Signal quality indices for the electrocardiogram and photoplethysmogram: Derivation and applications to wireless monitoring. IEEE Journal of Biomedical and Health Informatics.

[CR40] Pan, J., & Tompkins, W. J. (1985). A real-time QRS detection algorithm. IEEE Transactions on Biomedical Engineering, (3), 230–236. 10.1109/TBME.1985.325532 3997178

[CR41] Pantelopoulos A, Bourbakis NG (2010). A survey onwearable sensor-based systems for health monitoring and prognosis. IEEE Transactions on Systems Man, and Cybernetics, Applications and Reviews.

[CR42] Payne AFH, Schell AM, Dawson ME (2016). Lapses in skin conductance responding across anatomical sites: Comparison of fingers, feet, forehead, and wrist. Psychophysiology.

[CR43] Peirce JW (2009). Generating stimuli for neuroscience using PsychoPy. Frontiers in Neuroinformatics.

[CR44] Poh M-Z, Swenson NC, Picard RW (2010). A wearable sensor for unobtrusive, long-term assessment of electrodermal activity. IEEE Transactions on Biomedical Engineering.

[CR45] Reinhardt T, Schmahl C, Wüst S, Bohus M (2012). Salivary cortisol, heart rate, electrodermal activity and subjective stress responses to the Mannheim Multicomponent Stress Test (MMST). Psychiatry Research.

[CR46] Sartor F, Papini G, Cox LGE, Cleland J (2018). Methodological shortcomings of wrist-worn heart rate monitors validations. Journal of Medical Internet Research.

[CR47] Sawada Y, Tanaka G, Yamakoshi K i (2001). Normalized pulse volume (NPV) derived photo-plethysmographically as a more valid measure of the finger vascular tone. International Journal of Psychophysiology.

[CR48] Schubert, C., Lambertz, M., Nelesen, R. A., Bardwell, W., Choi, J. B., & Dimsdale, J. E. (2009). Effects of stress on heart rate complexity—a comparison between short-term and chronic stress. *Biological psychology, 80*(3), 325–332. 10.1016/j.biopsycho.2008.11.005 PMC2653595 19100813

[CR49] Shelley K, Shelley S, Lake CL, Hines RL, Blitt CD (2001). Pulse oximeter waveform: Photoelectric plethysmography. *Clinical monitoring practical applications for anesthesia and critical care*.

[CR50] Szalma JL, Hancock PA (2011). Noise effects on human performance: A meta-analytic synthesis. Psychological Bulletin.

[CR51] Torniainen, J., Cowley, B., Henelius, A., Lukander, K., & Pakarinen, S. (2015). Feasibility of an electrodermal activity ring prototype as a research tool. In *Proceedings of the Annual International Conference of the IEEE Engineering in Medicine and Biology Society, EMBS* (pp. 6433–6436). Piscataway, NJ: IEEE Press. 10.1109/EMBC.2015.7319865 10.1109/EMBC.2015.7319865 26737765

[CR52] Trull TJ, Ebner-Priemer U (2013). Ambulatory assessments. Annual Review of Clinical Psychology.

[CR53] van Dooren M, de Vries JJGGJ, Janssen JH (2012). Emotional sweating across the body: Comparing 16 different skin conductance measurement locations. Physiology and Behavior.

[CR54] Watson PF, Petrie A (2010). Method agreement analysis: A review of correct methodology. Theriogenology.

[CR55] Wilhelm P, Perrez M, Pawlik K, Conner TS, Mehl MR (2012). Conducting research in daily life: A historical review. *Handbook of research methods for studying daily life*.

[CR56] Wilke K, Martin A, Terstegen L, Biel SS (2007). A short history of sweat gland biology. International Journal of Cosmetic Science.

[CR57] Willemsen GHM, De Geus EJC, Klaver CHAM, Van Doornen LJP, Carroll D (1996). Ambulatory monitoring of the impedance cardiogram. Psychophysiology.

[CR58] Zaki R, Bulgiba A, Ismail R, Ismail NA (2012). Statistical methods used to test for agreement of medical instruments measuring continuous variables in method comparison studies: A systematic review. PLoS ONE.

[CR59] Zheng, Y., & Poon, C. C. Y. (2016). Wearable devices and their applications in surgical robot control and p-medicine. In *2016 IEEE 20th International Conference on Computer Supported Cooperative Work in Design (CSCWD)* (pp. 659–663). Piscataway, NJ: IEEE Press. 10.1109/CSCWD.2016.7566067

